# Early gestational prediction of spontaneous preterm birth using a validated three-protein serum biomarker panel

**DOI:** 10.1186/s12916-026-04639-9

**Published:** 2026-02-02

**Authors:** Qiong Luo, Juan Wei, Yun Ding, Yixuan Chen, Linlin Wu, C. James Chou, Xiaohua Luo, Negin Ghafourian, Jian Tao, Bo Jin, Kuo-Jung Su, Richard D. Mortensen, James Schilling, Zhi Han, Naoto Ozawa, Takumi Ichikawa, Ruben Y. Luo, Karl G. Sylvester, Scott R. Ceresnak, Ronald J. Wong, Lu Tian, Ivana Marić, Nima Aghaeepour, Brice Gaudilliere, Martin S. Angst, Gary M. Shaw, Doff McElhinney, Harvey J. Cohen, Gary L. Darmstadt, Jianmin Niu, David K. Stevenson, Xuefeng B. Ling

**Affiliations:** 1 Women’s Hospital, Zhejiang University School of Medicine, Hangzhou, 310006 China; 2 School of Medicine, Stanford University, Stanford, CA 94305 USA; 3 Shenzhen Maternity & Child Healthcare Hospital, Shenzhen, 518028 China; 4 Department of Reproductive Medicine Center, Henan Provincial People’s Hospital, Zhengzhou University People’s Hospital, Zhengzhou, 450003 Henan China; 5 The Eighth Affiliated Hospital, Sun Yat-Sen University, Shenzhen, 518033 China; 6 OncoOmicsDx Clinical Laboratory, Rockville, MD 20850 USA; 7 The Third Affiliated Hospital of Zhengzhou University, Zhengzhou, 450052 China; 8 Nippon Life Insurance Company, Osaka, 5410042 Japan

**Keywords:** Spontaneous preterm birth, Pregnancy, Serum biomarkers, Proteomics, And Risk prediction

## Abstract

**Background:**

Spontaneous preterm birth (sPTB) remains a major contributor to neonatal morbidity and mortality, with limited reliable early prediction tools. Existing biomarkers, such as the insulin-like growth factor-binding protein 4 (IBP4) to sex hormone-binding globulin (SHBG) ratio, offer modest predictive performance and are restricted to mid-gestation use (18–20 weeks), limiting their utility for timely intervention. We aimed to develop and validate a novel serological test based on early-gestational sampling to predict the risk of sPTB.

**Methods:**

We conducted a meta-analysis of 18 placental transcriptomic datasets to identify candidate genes associated with sPTB, resulting in 21 protein candidates tested by targeted proteomics. We developed a three-protein panel (glutathione peroxidase 3, GPX3; nidogen-1, NID1; and pappalysin-2, PAPPA2) and validated it in four independent cohorts (456 subjects and 1048 serum specimens) from the USA and Asia. Longitudinal serum samples were collected from 5 weeks and were analyzed using mass spectrometry and ELISA platforms. Predictor performance was compared to the IBP4/SHBG ratio.

**Results:**

The three-protein predictor (GPX3, NID1, and PAPPA2) demonstrated reproducible and superior performance across cohorts: *AUC* 0.74 (95% *CI* 0.59–0.88) in Alabama, 0.93 (95% *CI* 0.88–0.99) in California, 0.80 (95% *CI* 0.75–0.85) in Asia 1, and 0.83 (95% *CI* 0.70–0.95) in Asia 2. This outperformed the IBP4/SHBG ratio, which achieved AUCs of 0.68 (95% *CI* 0.50–0.89), 0.77 (95% *CI* 0.67–0.88), 0.59 (95% *CI* 0.52–0.65), and 0.61 (95% *CI* 0.50–0.75), respectively. Across obstetric trimesters, the three-protein panel maintained high predictive accuracy in the first and second trimesters (*AUROC* 0.82–0.97), the window when preventive interventions such as progesterone, cerclage, and low-dose aspirin are most effective. Kaplan–Meier analyses confirmed significantly earlier delivery among high-risk pregnancies identified by the three-protein panel.

**Conclusions:**

This maternal serum test provides a reliable approach for early risk assessment of sPTB. The three-protein panel demonstrated reproducible performance across cohorts and across PPROM-positive and PPROM-negative phenotypes, with the strongest discrimination in the first and second trimesters, when preventive therapies such as progesterone or cerclage are most effective. These findings support its potential as an early, clinically actionable screening tool for improving pregnancy outcomes.

**Supplementary Information:**

The online version contains supplementary material available at 10.1186/s12916-026-04639-9.

## Background

Spontaneous preterm birth (sPTB) refers to the spontaneous onset of labor or preterm premature rupture of membranes (PPROM) resulting in delivery before 37 completed weeks of gestation, distinguishing it from iatrogenic or medically indicated preterm deliveries undertaken for maternal or fetal complications [1, 2]. The prevalence of sPTB varies geographically, affecting approximately 12–13% of births in the USA and 5–9% in other developed countries, reflecting regional disparities and underlying complexities in etiology and risk exposure [3]. Infants born preterm face substantial short- and long-term complications, including respiratory distress, neurodevelopmental delay, and increased lifetime risk of cardiovascular disease and diabetes [4, 5]. In economic terms, the societal burden is profound: the 2016 US birth cohort incurred an estimated US $25.2 billion in excess costs due to prematurity, a figure expected to rise globally [6].

sPTB arises from a heterogeneous set of maternal and fetal risk factors, including a prior history of sPTB, chronic health conditions, environmental exposures, and genetic predisposition [3, 5, 7, 8]. These multifactorial contributors underscore the need for early and accurate risk assessment to enable preventive interventions. Current screening strategies, such as cervical length measurement and risk assessment based on obstetric history, lack sensitivity and fail to detect a substantial proportion of at-risk pregnancies [9]. This limitation has prompted efforts to develop biomarker-based tools that can improve predictive performance and offer broader population applicability [10].

Molecular studies have identified several biological pathways implicated in sPTB, including those related to inflammation, impaired trophoblast invasion, and aberrant angiogenesis [11–14]. For example, excessive Wnt signaling in the placenta has been associated with sPTB [14], and transcriptomic profiling has identified gene signatures linked to both early and late sPTB [15]. Similarly, cell-free RNA [16] and metabolomics-based markers [17, 18] have demonstrated promise in early prediction efforts. Despite these advances, few tests have been successfully translated into clinical use.

One commercially available test, PreTRM®, based on the ratio of insulin-like growth factor-binding protein 4 (IBP4) and sex hormone-binding globulin (SHBG) in maternal serum, uses mid-gestation samples, between 18 and 20^6/7^ weeks of gestation (126–146 days), to estimate sPTB risk. However, its predictive performance has been modest, with reported receiver-operating-characteristic area under the curve (ROC AUC) values of 0.67 in US validation studies and approximately 0.64 in South Asian and African populations [19, 20]. Importantly, the timing of this test limits its clinical impact, as it is performed relatively late in pregnancy, often too late for timely intervention. Early identification of women at increased risk before approximately 16–20 weeks’ gestation is clinically valuable because evidence-based preventive therapies are most effective when started early in pregnancy. For example, vaginal progesterone or cervical cerclage is recommended for women with a short cervix, ideally initiated between 14 and 24 weeks of gestation, and low-dose aspirin for preeclampsia prevention should begin before 16 weeks in high-risk individuals [21–24]. Earlier biomarker-based risk prediction could therefore complement existing cervical-length screening protocols, enabling timely initiation of these interventions and closer surveillance during the critical window when placental and cervical remodeling remain modifiable.

To overcome these limitations, our group has explored multi-omics approaches for sPTB prediction over the past decade [7, 25–28]. In the present study, we hypothesized that transcriptomic signatures from preterm placentas could identify circulating serum proteins predictive of sPTB early in gestation. By integrating placental gene expression data with targeted proteomic profiling and immunoassay validation, we developed a novel three-protein biomarker panel and evaluated its performance across four independent maternal cohorts from the USA and Asia. This study aims to assess the utility of this biomarker panel for early and robust prediction of spontaneous preterm birth.

## Methods

### Ethical approval

**Fig. 1:**
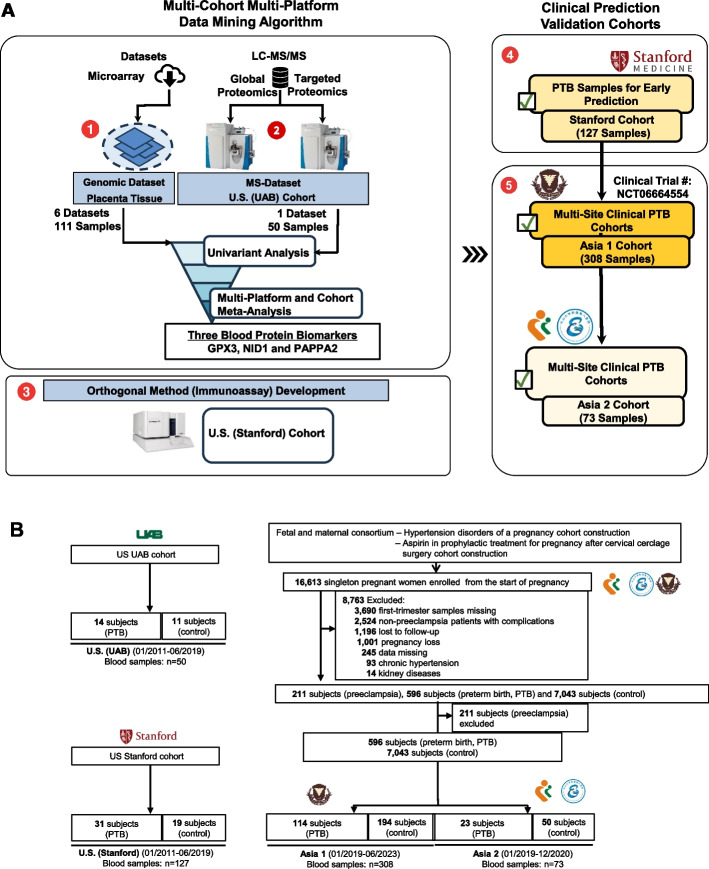
Overview of study design, cohort recruitment, and analytic sample selection. **A** Schematic of the multi-site prospective cohort enrollment, specimen collection, and nested case–control analytic workflow. **B** CONSORT-style diagram showing participant recruitment, exclusions, and analytic subset composition across four cohorts: US (UAB), US (Stanford), Asia 1, and Asia 2. The upper portion of each panel (e.g., 596 sPTB and 7043 controls) represents the total number of participants prospectively enrolled at each site in ongoing pregnancy registries, while the lower boxes (e.g., 114 sPTB + 194 term in Asia 1; 23 sPTB + 50 term in Asia 2) indicate the analytic subsets included in this biomarker study—those with available pre-outcome serum samples and complete clinical data. Within each cohort, all available spontaneous preterm birth (sPTB) cases and frequency-matched term controls were selected under blinded conditions to enable efficient biomarker evaluation. Consequently, the sPTB proportions in the analytic datasets reflect intentional case enrichment inherent to the nested case–control design and do not represent true population prevalence. This design ensures unbiased pre-outcome sampling and adequate statistical power while maintaining consistency across cohorts

As diagrammed in Fig. 1A, this study was approved by the Institutional Review Boards (IRBs) or ethics committees at each participating institution: University of Alabama at Birmingham: IRB no. F160906009; Stanford University: IRB no. 39264; Hangzhou Women's Hospital (Asia 1): IRB no. 2021–01–003; and Shenzhen Maternity and Child Health Hospital (Asia 2): IRB no. 2021–09-001. All participants were enrolled under IRB-approved protocols at each study site, and written informed consent was obtained prior to sample collection.

Data collection, processing, and sharing complied with the ethical principles of the Declaration of Helsinki and institutional data protection regulations. All clinical data were de-identified prior to analysis. Maternal serum samples were stored at − 80 °C and processed under IRB-approved protocols using coded identifiers. Mass spectrometry and ELISA experiments were conducted using blinded and randomized sample sets to ensure impartiality. No protected health information was shared outside the originating institutions.

### Study design and participants

This study employed a prospective, nested case–control design embedded within four independent pregnancy cohorts: a discovery cohort from the University of Alabama at Birmingham (UAB), a longitudinal validation cohort from Stanford University (California), and two external validation cohorts in China (NCT06664554) to assess cross-population applicability: Hangzhou Women’s Hospital (Asia 1) and Shenzhen Maternity and Child Health Hospital (Asia 2). The cohort-specific information is detailed in the CONSORT diagram (Fig. 1B). The US cohorts (Stanford and UAB) employed longitudinal serum collection, with participants providing serial samples across all three trimesters under standardized IRB-approved protocols. In contrast, the Asian cohorts were prospectively recruited with single-point serum collection during routine prenatal visits at tertiary hospitals. Because patients in these settings often receive antenatal care across multiple institutions, longitudinal follow-up was less frequent. For biomarker evaluation, we used a nested case–control design within each cohort, including all available sPTB cases and frequency-matched term controls, leading to case enrichment by design rather than by selective recruitment.

Inclusion criteria were harmonized across cohorts and included the following: maternal age ≥ 18 years, a singleton gestation, availability of at least one maternal serum sample collected between 5 and 36 weeks of gestation, and a known pregnancy outcome. Exclusion criteria comprised multiple gestation, major fetal anomalies, chronic hypertension, medically indicated preterm delivery (e.g., preeclampsia with severe features or fetal growth restriction), and incomplete clinical data. Outcome sPTB was defined as delivery before 37 weeks due to spontaneous preterm labor or preterm premature rupture of membranes (PPROM). To assess phenotype-specific performance, participants were categorized as PPROM + (delivery preceded by membrane rupture) or PPROM − (intact membranes with spontaneous labor). For sensitivity analyses, we further classified early sPTB as delivery < 34 weeks. All blood samples were collected before the onset of labor or membrane rupture to ensure pre-outcome biomarker assessment. Gestational age was assigned by a first-trimester ultrasound. For classification purposes, PPROM + cases included women who experienced spontaneous membrane rupture before 37 weeks, regardless of whether subsequent delivery occurred after spontaneous labor onset or following induction of labor (IOL). This definition follows ACOG guidance [29] that deliveries precipitated by spontaneous membrane rupture are classified within the spontaneous preterm birth spectrum. Medically indicated preterm deliveries (e.g., preeclampsia, fetal growth restriction, or elective iatrogenic preterm birth) were excluded.

For each site, the analytic population comprised all available spontaneous preterm birth (sPTB) cases with pre-outcome serum samples and a frequency-matched set of term controls drawn from the same prospective cohort. Thus, the total cohort sizes shown in Fig. 1 correspond to the larger registry populations, while the smaller numbers indicate the nested case–control subsets used for biomarker analysis. This approach ensured unbiased pre-outcome sampling while maintaining assay feasibility. The final analytic population included 456 participants and 1048 serum samples across all four cohorts. Demographic and clinical characteristics are summarized in Tables 1, 2, 3, and 4. Prevalence of sPTB ranged from 25% in the Stanford cohort to 37% in the Hangzhou cohort. Maternal age ranged from 28 to 33 years across sites, with parity and gestational age at blood sampling varying by cohort.

**Table 1:** Demographics of Alabama cohort (UAB)

	Term	sPTB	Test stat	*p*-value
Patient number	11	14		
Age, years			*t*-test (23 df) = 0.7	0.49
Mean (SD)	25.5 (5.1)	26.9 (4.7)		
BMI, kg/m^2^			*t*-test (22 df) = 0.58	0.57
Mean (SD)	28.1 (7.6)	30.2 (10.1)		
Race			Fisher’s exact test	0.487
White	0 (0)	2 (14.3)		
Black	11 (100)	11 (78.6)		
Other	0 (0)	1 (7.1)		
Category			Fisher’s exact test	< 0.001
Control	11 (100)	0 (0)		
PTB	0 (0)	14 (100)		
GA at delivery, weeks			Rank-sum test	< 0.001
Median (IQR)	37 (37, 38.5)	28 (26.2, 32)		
Mode of delivery			Fisher’s exact test	0.016
Cesarean delivery	0 (0)	6 (42.9)		
NSVD	11 (100)	7 (50.0)

**Table 2:** Demographics of California cohort (SU)

	Term	sPTB	Test stat	*p*-value
Patient number	19	31		
Age, years			*t*-test (48 df) = 0.11	0.915
Mean (SD)	31.9 (4.9)	31.7 (6.5)		
BMI, kg/m^2^			Rank-sum test	0.001
Median (IQR)	22.5 (20.5, 25.2)	28.2 (23.3, 32.2)		
Race			Fisher’s exact test	< 0.001
American Indian	0 (0)	2 (6.5)		
Asian-Thai	0 (0)	1 (3.2)		
Black	0 (0)	1 (3.2)		
Indian	0 (0)	1 (3.2)		
Pacific Islander	0 (0)	1 (3.2)		
White	19 (100)	13 (41.9)		
Other	0 (0)	12(38.8)		
Category			Chi-sq. (1 df) = 50	< 0.001
Term	19 (100)	0 (0)		
PTB	0 (0)	31 (100)		
GA at delivery, weeks			Rank-sum test	< 0.001
Median (IQR)	40 (39, 41)	34 (32, 36)		
Mode of delivery			Fisher’s exact test	< 0.001
Cesarean delivery	6 (31.6)	20 (64.5)		
NSVD	12 (63.2)	11 (35.5)		
OVD	1 (5.3)	0 (0)

**Table 3:** Demographics of Asia cohort 1

	Term	sPTB	Test stat	*p*-value
Patient number	194	114		
Age, years			Rank-sum test	< 0.001
Median (IQR)	30 (28, 32)	31 (29, 34)		
BMI, kg/m^2^			Rank-sum test	0.001
Median (IQR)	25.5 (23.8, 27.5)	24.5 (22, 26.6)		
Race			Chi-sq. (1 df) = 20.78	< 0.001
Asian	194 (100)	114 (100)		
Category			Chi-sq. (1 df) = 308	< 0.001
PTB	0 (0)	114 (100)		
Term	194 (100)	0 (0)		
GA at delivery, weeks			Rank-sum test	< 0.001
Median (IQR)	39 (39, 40)	35 (34.2, 36)		
Mode of delivery			Chi-sq. (1 df) = 22.08	< 0.001
Cesarean delivery	61 (31.4)	67 (58.8)		
NSVD	133 (68.6)	47 (41.2)

**Table 4:** Demographics of Asia cohort 2

	Term	sPTB	Test stat	*p*-value
Patient number	50	23		
Age, years			*t*-test (71 df) = 0.94	0.349
Mean (SD)	31.5 (4.8)	32.6 (3.8)		
BMI, kg/m^2^			Rank-sum test	0.976
Median (IQR)	20.4 (18.8, 22.7)	20.2 (19.2, 21.5)		
Race			Chi-sq. (1 df) = 9.99	0.002
Asian	50 (100)	23 (100)		
Category			Chi-sq. (1 df) = 73	< 0.001
Term	50 (100)	0 (0)		
PTB	0 (0)	23 (100)		
GA at delivery, weeks			Rank-sum test	< 0.001
Median (IQR)	39 (38, 40)	34 (32.5, 36)		
Mode of delivery			Chi-sq. (1 df) = 8.36	0.004
Cesarean delivery	14 (28.0)	17 (73.9)		
NSVD	25 (50.0)	6 (26.1)		

**Fig. 2:**
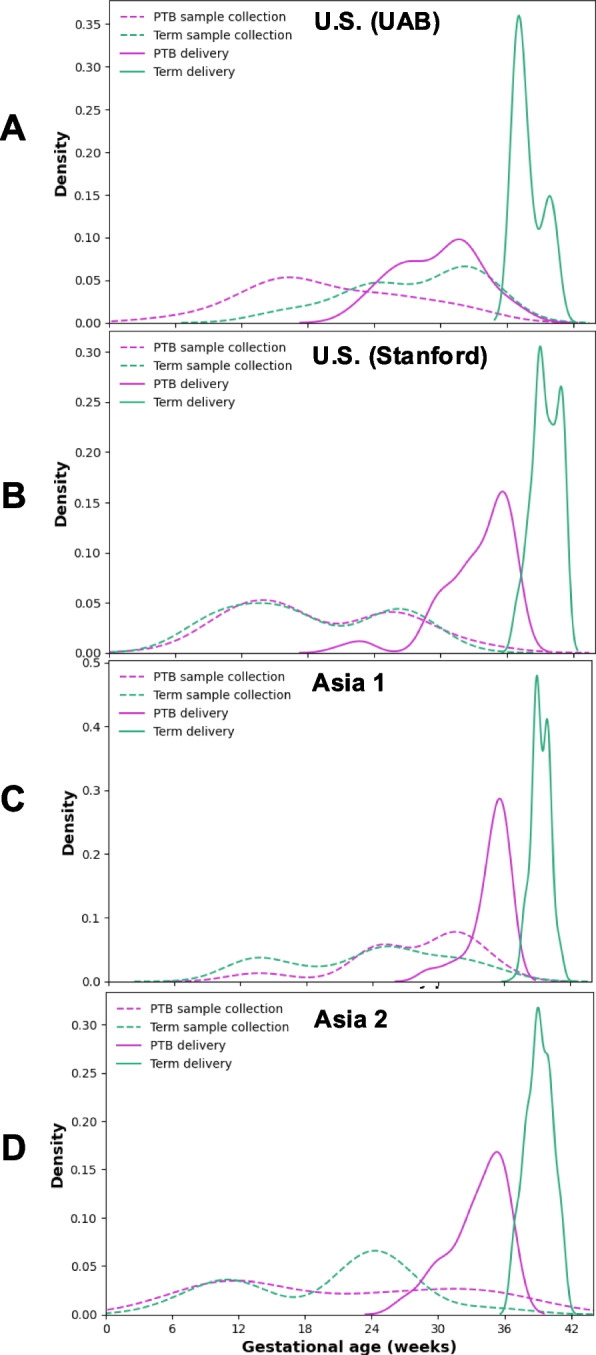
Gestational age distribution at sampling and delivery across cohorts. Density plots illustrate the gestational ages at sample collection (dashed lines) and delivery (solid lines) for women with sPTB (purple) and term pregnancies (green) across four cohorts: **A** US (UAB), **B** US (Stanford), **C** Asia 1, and **D** Asia 2

The UAB discovery cohort enrolled 25 participants, including 11 who delivered at term and 14 who experienced sPTB. Each participant contributed a single serum sample collected between 13 and 29 weeks of gestation. The Stanford validation cohort included 50 participants (19 term, 31 sPTB), who collectively provided 127 longitudinal serum samples collected between 7 and 36 weeks. Asia cohort 1 (Hangzhou) comprised 308 participants (194 term, 114 sPTB), contributing a total of 448 longitudinal samples between 5 and 30 weeks. Asia cohort 2 (Shenzhen) enrolled 73 participants (50 term, 23 sPTB), each providing a single serum sample collected between 5 and 28 weeks of gestation. Gestational age at sampling and delivery for all participants is shown in Fig. 2. The Stanford and Hangzhou cohorts enabled longitudinal assessment of biomarker dynamics through up to four samples per individual, whereas the UAB and Shenzhen cohorts contributed single samples per subject. Although longitudinal specimens extended to late gestation, the primary analysis focused on samples collected ≤ 24 weeks, corresponding to the window when preventive interventions are clinically actionable; later samples were analyzed descriptively to assess biomarker stability. Serum was collected using standard protocols: blood was drawn into serum separator tubes, allowed to clot for 30 min, and then centrifuged at 1500 × g for 10 min at 4 °C. Serum was aliquoted and stored at − 80 °C. All samples were randomized across analytical batches and processed under blinded conditions.

### Placental transcriptomic meta-analysis

**Fig. 3:**
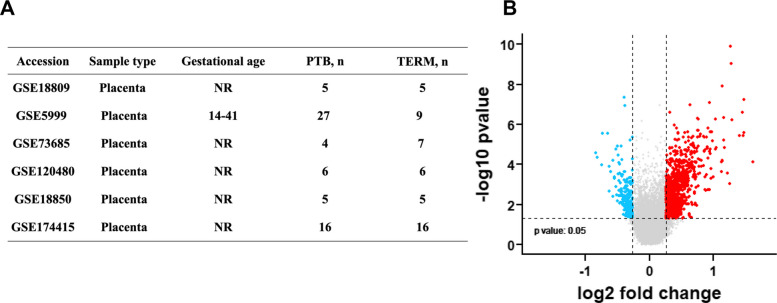
Meta-analysis of transcriptomic datasets: a volcano plot showing differentially expressed placental genes

To identify gene expression changes associated with sPTB, we performed a meta-analysis of placental transcriptomic datasets from the NCBI Gene Expression Omnibus (GEO) database. Eighteen datasets were initially screened using the keywords “preterm birth” and “placenta.” Datasets were eligible for inclusion if they (1) involved human placental tissue, (2) compared spontaneous preterm birth cases with term controls, and (3) provided raw or preprocessed gene expression data. Six datasets [30–40] met the inclusion criteria and were selected for meta-analysis (see Fig. 3A for an overview of the datasets).

To ensure consistency across datasets, all microarray data were normalized and batch-corrected prior to integration. Differential expression analysis was performed using the robust rank aggregation (RRA) framework [41], which integrates ranked gene lists across multiple studies while accounting for dataset-specific variability. Random-effects modeling was used to calculate meta-fold changes for each gene, incorporating both within- and between-study variances. Statistical significance was assessed using Fisher’s combined probability method.

Genes were retained as significant if they met the following criteria: (1) Were present in all six datasets, (2) had an absolute fold-change ≥ 1.2, and (3) had a meta *p*-value < 0.05. The meta-analysis revealed 926 upregulated and 200 downregulated genes in sPTB placentas compared with term controls (Fig. 3B). These differentially expressed genes were prioritized for downstream serum-based proteomic validation based on annotation in the UniProtKB/Swiss-Prot database and the presence of known or predicted secreted protein products. A full list of differentially expressed genes is provided in Additional File 1.

### Proteomic discovery and targeted validation

**Fig. 4:**
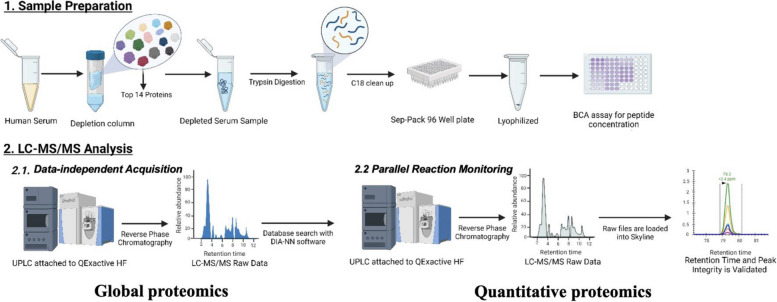
Parallel reaction monitoring (PRM) was used to validate TARGETED protein biomarker candidates

To translate placental transcriptomic signals into circulating sPTB biomarkers, we employed a two-stage proteomic workflow (Fig. 4) combining global data-independent acquisition (DIA) with targeted parallel reaction monitoring (PRM) mass spectrometry. All proteomic assays were conducted using harmonized protocols and blinded batch randomization.

Candidate refinement followed a multistage filtering pipeline (Table 5). Placental transcriptomic meta-analysis identified 1126 genes differentially expressed between preterm and term placentas. Global serum proteomics detected 530 unique proteins, 110 of which overlapped with the placental gene list and were measurable in maternal serum. Because serum proteomics has inherent sensitivity constraints, these 110 represent only a subset of placental-origin candidates—many transcripts likely encode proteins that are not released into circulation or fall below the detection threshold of untargeted LC–MS/MS. Targeted PRM assays were then developed for proteins with reliable peptide transitions and stable isotope-labeled internal standards; 21 proteins met these analytical criteria, while 89 could not be reproducibly quantified. Differential testing in the UAB discovery cohort identified three upregulated proteins (GPX3, NID1, PAPPA2; Mann–Whitney *U*-test, *FDR* < 0.05) that maintained concordant direction and magnitude of change in the Stanford validation cohort. These three markers were therefore advanced to model construction and external validation.

**Table 5:** Steps in refining the placenta biomarker candidates down to the three-protein serological PTB predictor

Number of analytes	Refinement: biomarker discovery and validation process
1126	Differentially expressed genes (*n* = 1126) were identified from microarray meta-analysis of term and PTB placentas
530	Global proteomics revealed 530 unique proteins detectable after depletion in maternal sera
110	110/1126 biomarker candidates were detectable in human pregnancy sera by global proteomics
21	21/110 biomarker candidates were quantifiable by PRM assays in human pregnancy sera
3	3/21 biomarker candidates were validated to be with statistical significance between term and preterm sera in two cohorts

#### Serum proteome discovery by DIA-MS

Global proteomic profiling was conducted on pooled maternal serum samples from the UAB discovery cohort. Serum was first depleted of the top 14 abundant proteins using High Select spin columns (Thermo Fisher Scientific), followed by denaturation with 0.2% RapiGest, reduction with 5 mM DTT, alkylation with 15-mM iodoacetamide, and overnight tryptic digestion. Peptides were desalted by solid-phase extraction, quantified by microBCA assay, and reconstituted in 0.1% formic acid.

DIA-MS was performed on a Thermo Q Exactive HF mass spectrometer coupled to a Waters ACQUITY UPLC M-Class nanoLC system. Peptides were separated on a C18 column using a 150-min gradient and analyzed using 10 m/z precursor isolation windows. A total of 530 unique serum proteins were identified (Additional File 2).

#### Candidate filtering and transcriptome-proteome integration

The 530 DIA-identified proteins were intersected with the 1126 differentially expressed placental genes from the transcriptomic meta-analysis (Additional File 1), yielding 110 overlapping candidates (Additional File 3). From these, 21 proteins were prioritized based on detectability, biological relevance, and peptide uniqueness for targeted quantification.

#### Targeted quantification by parallel reaction monitoring (PRM)

Targeted PRM-MS was performed on individual serum samples across all cohorts. Peptide concentration was normalized to 0.1 µg/µL, and 1 µg was injected per run. Proteotypic peptides (2–3 per protein) were selected based on prior DIA runs and DIA-NN spectral libraries, with scheduling and validation in Skyline.

PRM acquisition was conducted on the same Q Exactive HF instrument using 1.6 m/z isolation windows and stepped HCD collision energies (22, 26, 30 eV). MS2 resolution was set to 120,000, with an AGC target of 1e6 and max injection time of 190 ms.

#### Data processing and quality control

PRM data were analyzed using Skyline (v21.2.0.425). Fragment ion peak areas were extracted for 3–5 high-quality y- or b-ions per peptide. Peptides with > 20% missing values or poor reproducibility were excluded. Peak area signals were log_2_-transformed, normalized using global median normalization, and z-scored within the cohort.

Final protein abundance values were computed by averaging replicate peptides. Analytical reproducibility was confirmed by technical replicates (coefficient of variation < 15%) and linearity in dilution series. Differential expression between sPTB and term samples was assessed using Mann–Whitney *U*-tests in the UAB cohort, and significantly upregulated proteins were selected for model development and validation.

### Immunoassay (ELISA) validation and cross-platform comparison

To confirm the cross-platform reproducibility and clinical utility of the selected protein biomarker panel, we performed orthogonal validation using commercial enzyme-linked immunosorbent assays (ELISAs). This validation was conducted in the Stanford cohort, in which longitudinal serum samples and matching LC–MS/MS data were available.

#### ELISA assay procedures

ELISA kits for GPX3, NID1, and PAPPA2 were purchased from validated commercial vendors (GPX3: Cloud-Clone Corp., SEA663Hu; NID1: MyBioSource, MBS2507399; PAPPA2: R&D Systems, DY2348-05). Assays were performed according to the manufacturers’ instructions. All serum samples were thawed on ice and diluted to the optimal concentration range for each assay, as determined by pilot standard curve titrations.

Samples and standards were run in duplicate. Absorbance was read at 450 nm using a BioTek Synergy HTX plate reader, and background correction was performed at 570 nm. Standard curves were generated using a four-parameter logistic regression model. Concentrations were interpolated and log-transformed for downstream analyses. Intra-assay and inter-assay coefficients of variation (CV) were maintained below 15%.

#### Cross-platform comparison

To assess concordance between ELISA and LC–MS/MS quantification, we compared the z-score-normalized abundance values of each protein across both platforms using Pearson correlation and Bland–Altman analysis. Only samples with matched ELISA and PRM data were included (*n* = 50 participants; 127 serum samples).

### Statistical analysis

All statistical analyses were performed using R (v4.2.1) and Python (v3.9) with the scikit-learn, statsmodels, and matplotlib packages. Analyses were conducted separately for each cohort and stratified by gestational age. A two-sided *p*-value < 0.05 was considered statistically significant unless otherwise noted.

Stratified analyses were performed when clinically or biologically justified. Gestational-age bins (< 16 16–20, > 20 weeks) and trimester groupings (T1 ≤ 13^+6^, T2 14–27^+6^, T3 ≥ 28 weeks) were defined a priori to reflect key windows for biomarker kinetics and intervention timing. Additional stratifications were performed by pathophysiology (PPROM + vs. PPROM** −**) and by clinical severity (delivery < 37 weeks vs. < 34 weeks) to assess model robustness across distinct subtypes of spontaneous preterm birth. Stratification was implemented only when subgroup sample sizes were sufficient (*n* ≥ 15 per stratum).

#### Data normalization and batch adjustment

 Protein abundance values were first adjusted using multiples of the median (MoM), by expressing each measurement relative to the gestational age–specific median within the relevant reference set to account for expected physiologic changes across pregnancy. MoM-adjusted values were then log-transformed and subsequently standardized within each cohort using z-scores (mean 0, SD 1) to harmonize scale across cohorts and platforms. All samples were randomized across LC–MS/MS and ELISA assay batches and analyzed blinded to the outcome. No batch effects were detected after normalization.

#### Univariate comparisons

Differences in individual protein levels between sPTB and term deliveries were assessed using the nonparametric Mann–Whitney *U*-test. Visualizations included volcano plots, violin plots, and boxplots for the three-protein panel (GPX3, NID1, PAPPA2) and for the comparator proteins (IBP4, SHBG).

#### Predictive composite score construction

The three-protein PTB predictor panel (GPX3, NID1, PAPPA2) was constructed by summing the three protein z-scores to generate a composite risk score, which was evaluated and compared against the established IBP4/SHBG expression ratio predictor. Separate PTB risk scores were computed using LC–MS/MS or ELISA input features, where applicable. Cross-validation with fivefold stratified random sampling was performed to evaluate prediction stability. To assess the clinical utility of the three-protein panel or the IBP4/SHPG ratio in stratifying patients by time to delivery, we applied a binary classification threshold derived from the optimal Youden index in the discovery cohort. This threshold was then applied across all validation cohorts to separate participants into predicted high-risk and low-risk groups for the sPTB outcome.

#### Performance evaluation

Predictor performance was assessed using receiver operating characteristic (ROC) curve analysis. Area under the curve (AUC) was used as the primary performance metric, with 95% confidence intervals calculated by nonparametric bootstrapping (1000 replicates). Sensitivity, specificity, positive predictive value (PPV), and negative predictive value (NPV) were calculated at the optimal threshold defined by the Youden index.

#### Longitudinal and stratified analyses

Gestational age-specific performance was evaluated by binning samples into three windows: < 16 weeks, 16–20 weeks, and > 20 weeks. ROC AUCs were computed within each interval. Additionally, our protein panel was applied to longitudinal profiling in the U.S. and Asia cohorts, allowing evaluation of temporal trends in risk prediction.

To account for differences in sPTB prevalence across cohorts and reflect real-world use cases, we applied prevalence-weighted bootstrapping to estimate PPV and panel score distributions. Classification probabilities for both our protein panel and IBP4/SHBG ratio were analyzed, before and after prevalence adjustment.

#### Survival analysis

The time to delivery was analyzed using Kaplan–Meier survival curves, comparing high- and low-risk pregnancies based on the predictive panel output. Group differences were evaluated using the log-rank test.

#### Sensitivity analyses

Sensitivity analyses were conducted to evaluate panel robustness across race and ethnicity, sampling time points, and individual proteins. Alternative classifiers (random forests, support vector machines) were evaluated but did not significantly improve performance.

#### Prevalence-weighted bootstrap for real-world PPV estimation

To approximate real-world deployment across populations with differing spontaneous preterm birth (sPTB) prevalence, we applied prevalence-weighted bootstrapping using site-specific prevalence priors derived from published epidemiologic data (10.4% for the U.S. cohorts, 5.9% for Asian 1 and 2). Each cohort’s analytic set was resampled 1000 times with replacement to reflect these population prevalences, and PPV and NPV were recalculated for each iteration.

## Results

### Biomarker discovery and refinement (Table 5)

We first conducted a placental transcriptomic meta-analysis to identify candidate genes associated with the sPTB outcome. Ten publicly available datasets comprising 759 placental tissue samples (365 sPTB and 394 term) were included in a pooled differential expression analysis. After false discovery rate (FDR) correction (*q* < 0.05), 1126 genes were identified as differentially expressed in sPTB compared with term controls, including 926 upregulated and 200 downregulated genes (Fig. 3). These genes were enriched for pathways related to inflammation, extracellular matrix remodeling, and angiogenesis, which are consistent with the biological processes implicated in premature labor. The complete gene list is provided in Additional File 1.

Next, to identify serum-accessible protein biomarkers, we performed data-independent acquisition (DIA) mass spectrometry on pooled serum from the UAB discovery cohort. A total of 530 unique proteins were identified in maternal serum (Additional File 2). These were intersected with the placental transcriptomic signatures to yield 110 candidate proteins that were both differentially expressed at the transcript level and detectable in maternal serum (Additional File 3).

From this set of 110 candidates, 21 proteins were prioritized for targeted validation based on uniqueness of tryptic peptides, detectability, and biological plausibility. These included known regulators of angiogenesis (FLT1, FLT4), placental invasion (LEP, PAPPA2), and immune signaling (GPX3, VCAM1).

**Fig. 5:**
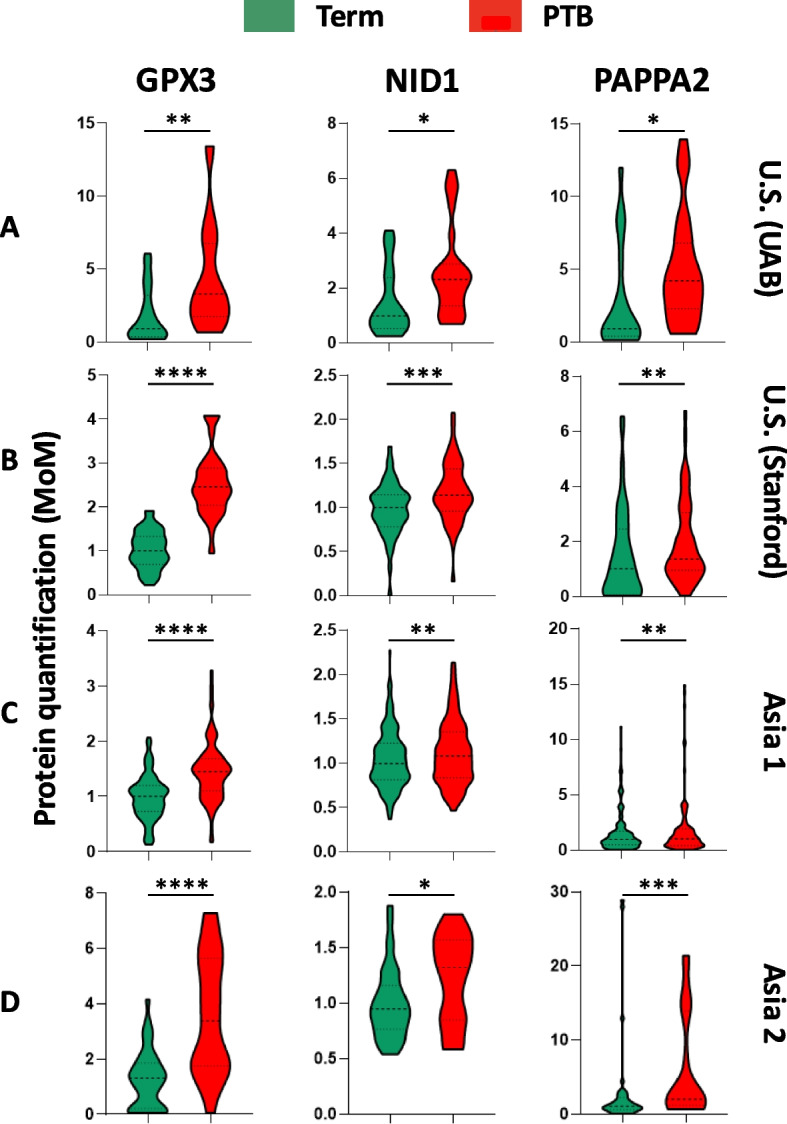
Expression levels of GPX3, NID1, and PAPPA2 in sPTB versus term pregnancies. This figure shows the quantification of three protein biomarkers, GPX3, NID1, and PAPPA2, in maternal serum samples, comparing term (green) and sPTB (red) pregnancies across four cohorts: **A** US (UAB), **B** US (Stanford), **C** Asia 1, and **D** Asia 2. Protein expression levels are expressed as MoM. Statistical significance is denoted by asterisks, where *p* is * < 0.05, ** < 0.01, *** < 0.001, and **** < 0.0001

Univariate analysis of the 21 proteins in the UAB cohort revealed that three proteins, glutathione peroxidase 3 (GPX3), nidogen-1 (NID1), and pappalysin-2 (PAPPA2), were consistently elevated in women who later delivered preterm. These three proteins were selected as the final predictive biomarker panel for further validation in independent cohorts. Their differential expression is shown in Fig. 5. The remaining 18 proteins showed inconsistent or nonsignificant trends and were therefore excluded from predictive panel construction.

### Cross-platform validation by ELISA

**Fig. 6:**
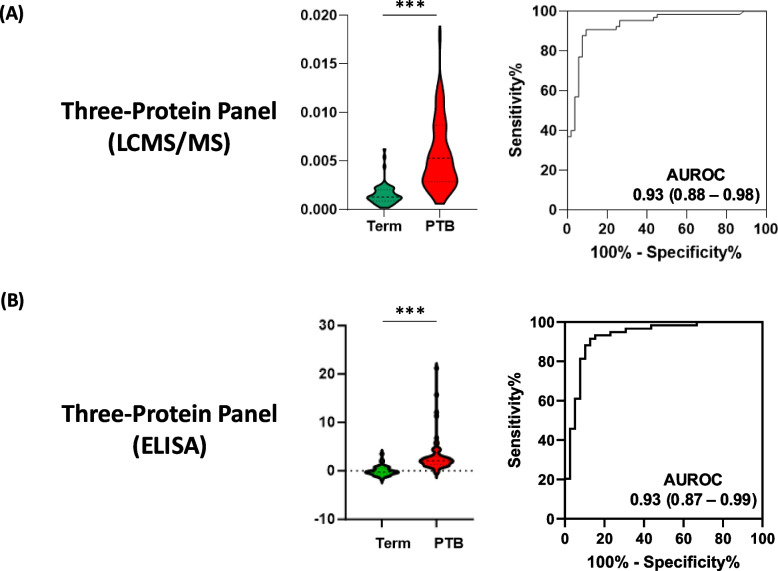
Orthogonal immune method validating PTB serological markers in the Stanford cohort. Comparison of the LCMS/MS-quantified three-protein panel (**A**) and the ELISA-quantified three-protein panel (**B**) for classifying term (green) and preterm birth (PTB, red) pregnancies. Violin plots show the distribution of predictor scores in term and PTB samples. The receiver operating characteristic (ROC) curves and area under the curve (AUC) values for the three-protein predictor show classification performance. LCMS/MS, liquid chromatography tandem mass spectrometry (MS); ELISA, enzyme-linked immunosorbent assay. ***p < 0.001

To evaluate the technical robustness and translational potential of the three-protein panel, we conducted cross-platform validation using commercial ELISA assays for GPX3, NID1, and PAPPA2. This biomarker panel analysis was performed in the Stanford cohort, where matched serum samples had been previously analyzed by LC–MS/MS (Fig. 6A) and subsequently by ELISA (Fig. 6B).

Protein concentration measurements obtained via ELISA were log-transformed and standardized. Pearson correlation analysis between ELISA and LC–MS/MS measurements showed strong concordance for all three proteins: GPX3 (*R* = 0.78), NID1 (*R* = 0.65), and PAPPA2 (*R* = 0.81), indicating consistent quantification across platforms.

We next constructed the predictive panel using ELISA-based input values and evaluated its performance in the Stanford cohort. The ELISA-based panel achieved an AUC of 0.93 (95% *CI* 0.87–0.99), closely mirroring the performance of the LC–MS/MS panel (*AUC* 0.93, 95% *CI* 0.88–0.98) in the same cohort. Stratified performance by gestational age window and predicted risk group also remained consistent, supporting the feasibility of transitioning the biomarker panel to lower-cost immunoassay platforms for broader clinical implementation.

Together, these findings demonstrate that the three-protein signature is analytically robust across both LC–MS/MS and ELISA platforms, and readily compatible with standard diagnostic workflows, providing a scalable and practical path toward clinical implementation.

### Predictive performance of the three-protein panel

**Fig. 7:**
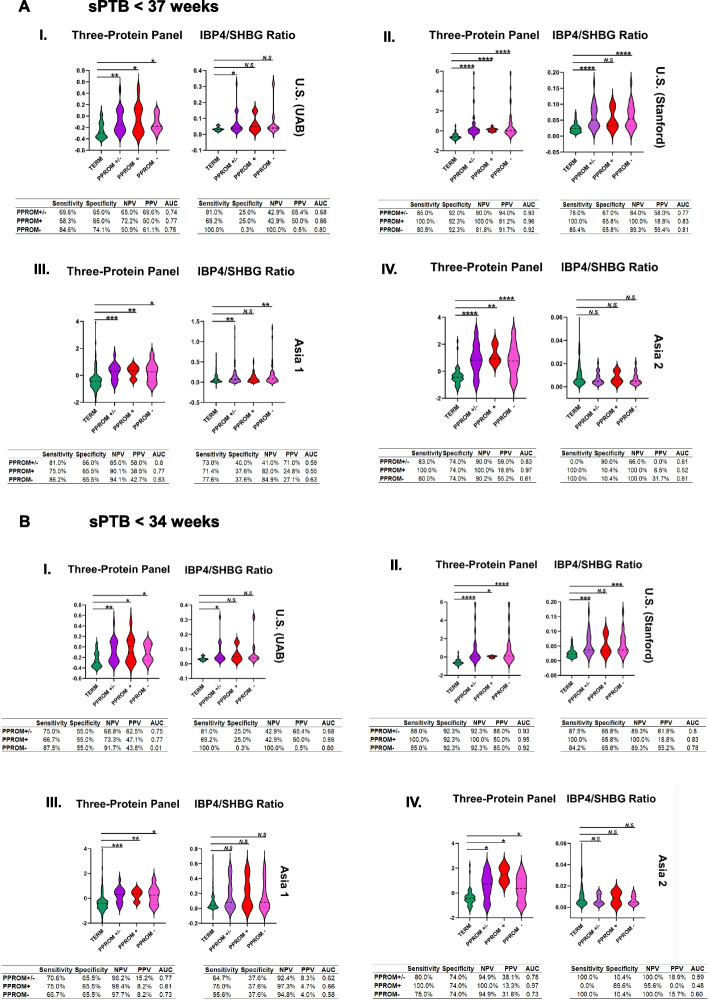
Performance of the three-protein panel by pathophysiology and gestational-age threshold. **A** Predictive performance for spontaneous preterm birth (< 37 weeks), stratified by PPROM + (premature rupture of membranes), PPROM − (spontaneous labor with intact membranes), and combined (“all sPTB”) groups. **B** Parallel analysis for early preterm birth (< 34 weeks). Tables below each cohort display AUC ROC, sensitivity, specificity, PPV, and NPV for the three-protein panel (GPX3, NID1, PAPPA2) and the IBP4/SHBG ratio comparator. All specimens were collected prior to labor or membrane rupture. Comparable performance across PPROM + and PPROM − groups indicates that the biomarker panel captures core pathways common to both spontaneous-labor and membrane-rupture etiologies, with stronger discrimination in earlier gestational windows relevant for clinical intervention. Asterisks denote statistical significance based on the Mann–Whitney *U*-test: *p < 0.05, **p < 0.01, ***p < 0.001. NS, not significant (*p* > 0.05)

We next evaluated whether biomarker performance differed between PPROM + and PPROM − phenotypes and whether predictive accuracy changed when restricting to earlier preterm thresholds. As summarized in Fig. 7A (deliveries < 37 weeks) and Fig. 7B (deliveries < 34 weeks), the three-protein panel (GPX3, NID1, PAPPA2) demonstrated comparable discriminative performance across both subtypes within each cohort. Positive and negative predictive values were similar between the PPROM + and PPROM − groups, suggesting that the biomarker signal reflects shared upstream biological processes rather than membrane-rupture-specific events. When benchmarked against the IBP4/SHBG ratio, the three-protein panel consistently achieved higher AUROC values and improved sensitivity across all cohorts. For *sPTB* < 37 weeks, the three-protein panel outperformed the IBP4/SHBG ratio in both PPROM + and PPROM − subgroups, with median AUROC differences of 0.12 to 0.25 (see Fig. 7A). This advantage persisted when restricting to early *sPTB* < 34 weeks, where the three-protein panel retained robust discrimination (*AUROC* 0.82–0.93) compared with the lower and more variable performance of the IBP4/SHBG ratio (*AUROC* 0.59–0.78, Fig. 7B). These findings indicate that the three-protein panel is particularly effective for identifying pregnancies at the highest risk for severe, early preterm delivery, irrespective of the presence or absence of PPROM. All serum samples were obtained prospectively before the onset of labor or membrane rupture, ensuring that measured differences reflect predictive rather than reactive changes in protein abundance. In Fig. 7, asterisks denote statistical significance from the Mann–Whitney *U*-test (*p < 0.05, **p < 0.01, ***p < 0.001, NS — not significant, *p* > 0.05). PPV and NPV values shown in Fig. 7 represent case-enriched analytic estimates derived from the nested case–control design.

**Fig. 8:**
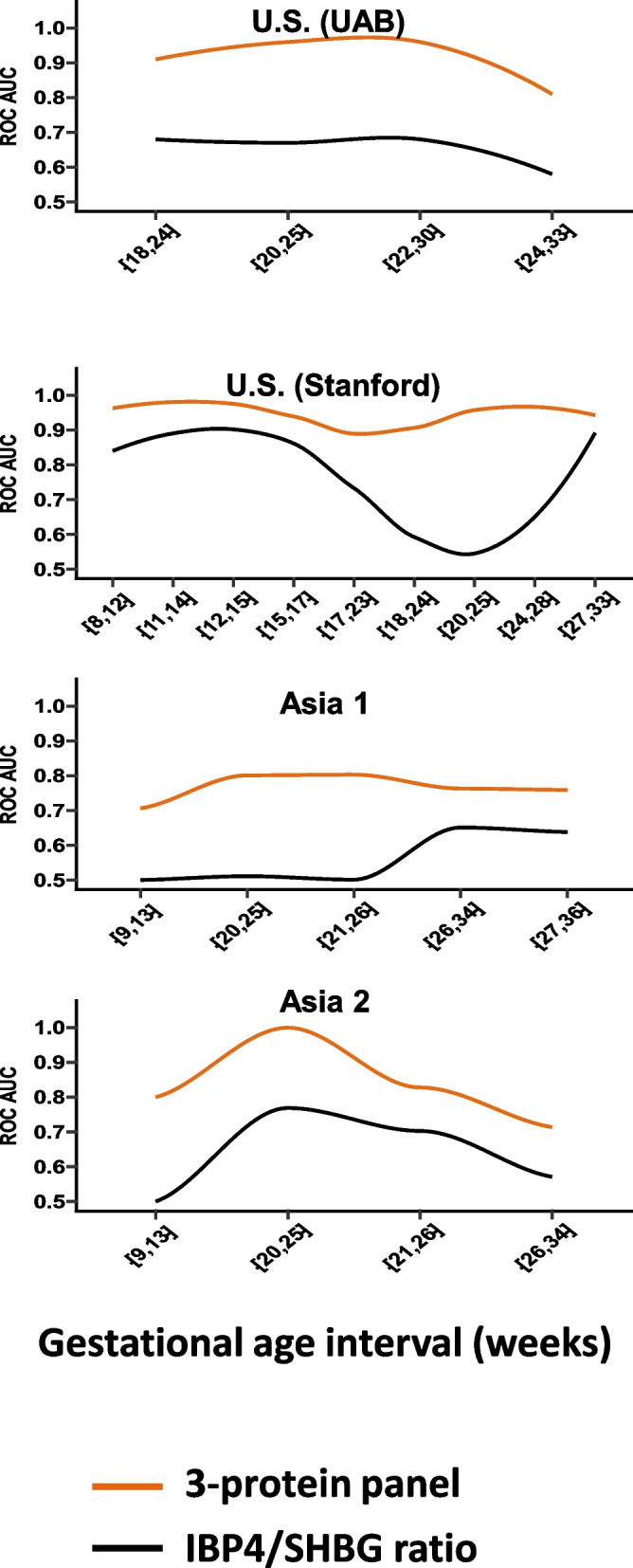
Performance of the three-protein panel and IBP4/SHBG across gestational-age bins. Receiver-operating characteristic (AUROC) values for the three-protein predictor (GPX3, NID1, PAPPA2; orange line) and the comparator IBP4/SHBG ratio (black line) across gestational-age bins (< 16 weeks, 16–20 weeks, > 20 weeks) in four cohorts: US (UAB), US (Stanford), Asia 1, and Asia 2. Per-bin sample numbers are provided in Additional File 4. Error bars indicate 95% bootstrap confidence intervals

To assess the robustness and clinical applicability of the biomarker panel across gestation, we first evaluated predictive performance in predefined gestational-age bins. Figure 8 shows the AUROC values for the three-protein panel (GPX3, NID1, PAPPA2) and the comparator IBP4/SHBG ratio across the < 16-week, 16–20-week, and > 20-week intervals in all four cohorts. The three-protein panel maintained strong discrimination across these windows, with cohort-specific AUROC values ranging from 0.74 to 0.93. Per-bin sample numbers are annotated in the figure and detailed in Additional File 4.

To further evaluate model behavior in clinically meaningful time frames, we examined predictive performance by obstetric trimester. Table 6 summarizes the AUROC (95% CI) values of the three-protein panel and the IBP4/SHBG ratio across cohorts. The three-protein panel achieved high accuracy during the first and second trimesters (*AUROC* 0.82–0.97), corresponding to the period when preventive interventions such as vaginal progesterone, cerclage, or low-dose aspirin are most effective and retained acceptable discrimination in the third trimester (*AUROC* 0.62–0.87). Per-stratum sample numbers are provided in Additional File 4.

**Table 6:** Trimester-specific predictive performance of the three-protein panel, summarizing the AUROC (95% CI) of the three-protein panel for spontaneous preterm-birth prediction with samples grouped by obstetric trimester: T1 (≤ 13^+6^ weeks), T2 (14–27^+6^ weeks), and T3 (≥ 28 weeks). Sample numbers for each stratum are provided in Additional File 4. 95% CIs were estimated using DeLong/bootstrapping. In trimester-stratified analyses with limited sample sizes, confidence intervals may span 0.50

Trimester	Biomarker panel	US (UAB)	US (Stanford)	Asia 1	Asia 2
1st	Three-protein predictor	/	0.97 (0.93, 1.00)	0.71 (0.42, 0.99)	0.77 (0.57, 0.97)
	IBP4/SHBG predictor	/	0.88 (0.72, 1.00)	0.43 (0.10, 0.78)	0.54 (0.30, 0.77)
2nd	Three-protein predictor	0.90 (0.78, 1.00)	0.92 (0.85, 0.99)	0.82 (0.75, 0.90)	0.83 (0.49, 1.00)
	IBP4/SHBG predictor	0.78 (0.60, 0.97)	0.72 (0.55, 0.88)	0.51 (0.41, 0.61)	0.70 (0.49, 0.91)
3rd	Three-protein predictor	0.71 (0.45, 0.97)	0.87 (0.67, 1.00)	0.72 (0.63, 0.82)	0.62 (0.15, 1.00)
	IBP4/SHBG predictor	/	0.93 (0.76, 1.00)	0.70 (0.61, 0.79)	0.76 (0.42, 1.00)

**Fig. 9:**
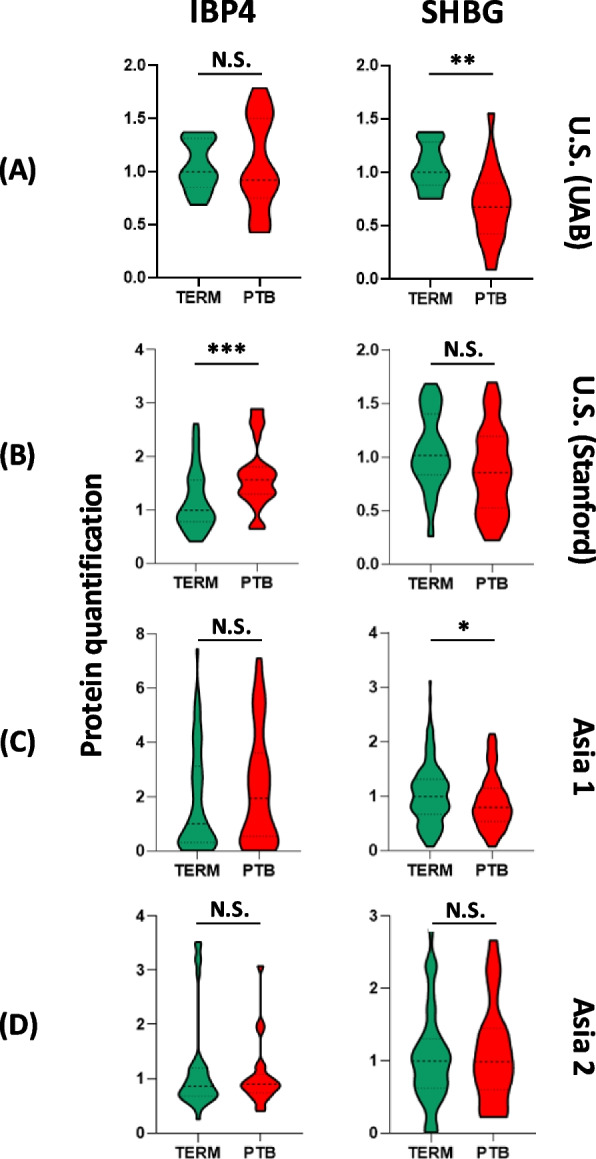
IBP4 and SHBG protein expression level analysis. The results illustrate the distribution of IBP4 (left) and SHBG (right) expression levels for samples with either term (green) or sPTB (red) outcomes. Statistical significance is denoted by asterisks, where *p* is * < 0.05, ** < 0.01, *** < 0.001, and **** < 0.0001. NS, not significant. *p* > 0.05

To contextualize these findings, we also examined the serum expression of IBP4 and SHBG directly. As shown in Fig. 9, IBP4 levels were mildly elevated in sPTB across cohorts, whereas SHBG exhibited inconsistent expression trends, particularly in the Asian cohorts. These biological differences likely contributed to the poorer performance of the IBP4/SHBG ratio model outside its original development population.

The variable gestational-age distributions across cohorts, although superficially uneven, mirror real-world conditions in which sample timing varies with healthcare access and delivery timing. As shown in Fig. 2, sampling gestational ages varied between term and preterm groups across sites, reflecting differences in local clinical workflows and sample-collection schedules. At UAB, longitudinal collections naturally ended earlier for women who delivered preterm, whereas in Asia 2 most participants contributed a single mid- to late-pregnancy sample. Despite these inherent differences, the three-protein panel maintained consistent discriminative performance across gestational-age bins and trimesters (Figs. 7 and 8, Table 6), demonstrating the model’s robustness to real-world variability in sampling timing.

Overall, the three-protein panel provided more accurate and earlier sPTB risk prediction across diverse cohorts and gestational timepoints than the current IBP4/SHBG-based comparator.

### Time to delivery and risk group separation

**Fig. 10:**
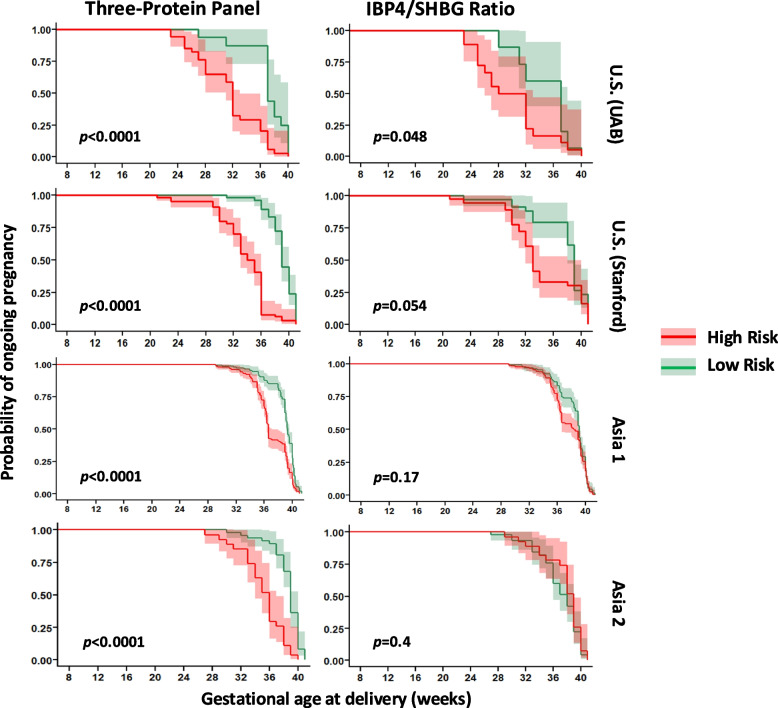
Kaplan–Meier analysis comparing delivery timing between predicted risk groups. Kaplan–Meier curves display the probability of ongoing pregnancy as a function of gestational age at delivery (*x*-axis) for pregnancies classified as high-risk (red) or low-risk (green) for sPTB. Risk stratification was conducted using the three-protein predictor (left panels) and the IBP4/SHBG ratio (right panels) across four cohorts: US (UAB), US (Stanford), Asia 1, and Asia 2

Kaplan–Meier survival analysis demonstrated a significant separation in gestational age at delivery between predicted high-risk and low-risk groups. As shown in Fig. 10 (left panel), participants classified as high risk by the three-protein panel delivered significantly earlier than those classified as low risk in all three cohorts (log rank *p* < 0.0001 for each site). The median time to delivery among high-risk participants was 3.2–5.1 weeks shorter than among low-risk participants, depending on the cohort.

In contrast, the IBP4/SHBG ratio model demonstrated poorer separation. As shown in Fig. 10 (right panel), although high-risk individuals, as defined by the IBP4/SHBG score, tended to have earlier deliveries on average, the survival curves overlapped substantially, particularly in the Asian cohorts, and log-rank tests were not statistically significant in two of the three validation sets.

These results indicate that the three-protein panel not only predicts sPTB with greater accuracy but also stratifies participants by clinically meaningful differences in delivery timing—information that could guide clinical decision-making and intervention timing.

### Risk score distribution and real-world prevalence

To examine the classification behavior of both the three-protein panel and the IBP4/SHBG model before and after real-world prevalence adjustment, we plotted the distribution of predicted risk scores across the Stanford, Asia 1, and Asia 2 cohorts. These distributions illustrate how each model separates sPTB and term outcomes and whether risk scores cluster clearly around a decision boundary.

**Fig. 11:**
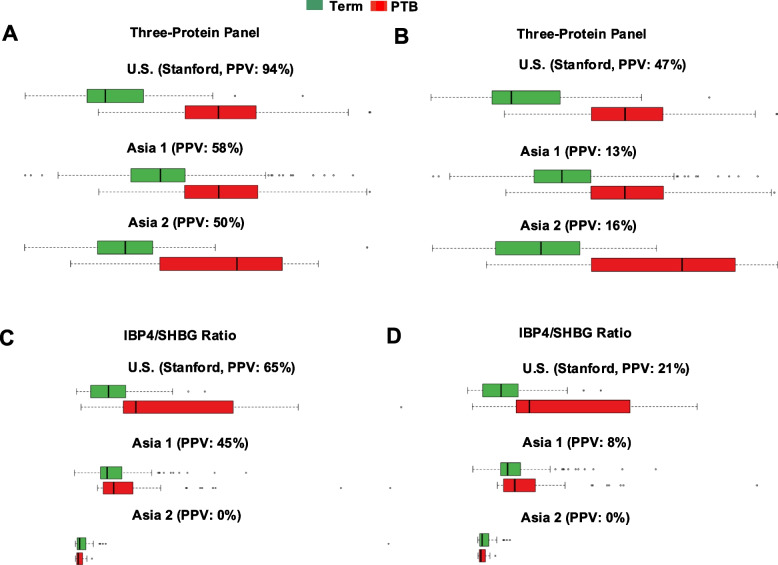
Risk score distributions and prevalence-adjusted PPV for the three-protein model and the comparator IBP4/SHBG ratio. PPVs and NPVs in Fig. 11 are prevalence-weighted using site-specific prevalence assumptions. For clarity, Fig. 7 presents case-enriched analytic PPVs/NPVs from the nested case–control subsets, whereas Fig. 11 presents prevalence-weighted estimates approximating real-world screening. The three-protein panel showed the classification score value distribution before (**A) **or after (**B**) bootstrapping, to reach population sPTB prevalence of 10.4% in the US (Stanford) and 5.9% in Asia, stratified by the samples in the California, Asia 1, and Asia 2 cohorts. The IBP4/SHBG ratios showed the classification score value distribution before (**C**) or after (**D**) bootstrapping, to reach population sPTB prevalence of 10.4% in the US (Stanford) and 5.9% in Asia 1 and Asia 2, stratified by the samples in the US (Stanford), Asia 1, and Asia 2 cohorts

As shown in Fig. 11A and B (real-world prevalence-adjusted), the three-protein panel produced well-separated score distributions for cases with term and sPTB outcomes, with minimal overlap. The model maintained a clear discrimination margin across cohorts and sampling windows. In contrast, the IBP4/SHBG model yielded overlapping score distributions with substantially reduced discriminatory power [Fig. 11C and D (real-world prevalence-adjusted)], consistent with its lower AUC.

Because the analytic sets use a nested case–control sampling of the underlying prospective cohorts, the observed sPTB proportions (25–37%) reflect case enrichment rather than population prevalence. To simulate deployment in real-world settings with varying prevalence of spontaneous preterm birth across populations, we applied prevalence-weighted bootstrapping to estimate positive predictive values (PPVs). The three-protein panel achieved adjusted PPVs of 13–47% across the validation cohorts (Fig. 11C). By contrast, the IBP4/SHBG ratio showed modest PPVs of 0–21% (Fig. 11D), only marginally better than the baseline prevalence.

Prevalence-weighted PPVs (Fig. 11B and D) provide an approximation of expected real-world predictive performance, whereas the higher PPVs observed in Fig. 7 and Fig. 11A and C reflect the analytic case-enriched subsets used for biomarker validation. These findings underscore the real-world clinical relevance of the three-protein panel, as it not only maintains high discriminative accuracy but also generates actionable risk stratification that could support individualized interventions, even in populations with relatively low sPTB prevalence.

## Discussion

Spontaneous preterm birth (sPTB), particularly when it occurs early in gestation, remains a major global contributor to neonatal morbidity and mortality. The ability to predict sPTB early in pregnancy, before clinical symptoms emerge, enables timely interventions and improved neonatal outcomes. Despite long-standing efforts, most pregnancies destined to end preterm are not identified using existing clinical tools. A prior history of sPTB and second-trimester cervical length remain the most reliable risk factors currently used in clinical practice [3, 42–44]. However, these indicators miss most cases and lack utility for first-time mothers. Multiple candidate biomarkers, including fetal fibronectin, placental alpha-microglobulin-1, and phosphorylated IGFBP-1, have been proposed [45, 46], yet even in combination, their predictive performance remains suboptimal [47].

A robust screening tool should be minimally invasive, highly predictive early in pregnancy, and easily integrated into routine prenatal care. Building on the foundation of prior “omics” studies, including proteomic [48–50], transcriptomic [30, 51], genomic [52, 53], and metabolomic [54, 55] efforts, we designed a biologically grounded, technically reproducible biomarker panel and validated it across diverse populations. This study represents a significant step forward in that direction. Using a combined transcriptomic meta-analysis and proteomic refinement strategy, we identified a three-protein panel, GPX3, NID1, and PAPPA2, that reliably predicts spontaneous preterm birth (sPTB), even in the late first and early second trimesters. The three-protein panel was constructed on the discovery cohort and externally validated across three independent cohorts in the USA and Asia, demonstrating reproducible rather than uniform performance (AUC range 0.74–0.93). Importantly, the panel was validated using both LC–MS/MS and ELISA platforms, enhancing its translational potential.

Compared to the existing IBP4/SHBG ratio test, previously developed using mass spectrometry [19], our three-protein panel achieved higher accuracy across all validation cohorts and gestational windows. In the Stanford cohort, the AUC reached 0.93 for our panel versus 0.77 for IBP4/SHBG; in Asia 1, 0.80 versus 0.59; and in Asia 2, 0.83 versus 0.61. Moreover, prevalence-adjusted positive predictive values (PPV) for our model were consistently superior, reaching up to 47/13/16% in the Stanford/Asia 1/Asia 2 cohorts and significantly outperforming the comparator IBP4/SHBG model of 21/8/0%. These findings are notable given that IBP4/SHBG is used clinically and typically administered at 19–20 weeks, often too late to initiate early interventions such as low-dose aspirin, progesterone, or lifestyle modification.

Each of the three proteins in our panel plays a biologically plausible role in pregnancy maintenance. PAPPA2, a placenta-derived metalloproteinase, regulates the bioavailability of insulin-like growth factors (IGFs) by cleaving IGF-binding proteins, thereby modulating fetal growth and placental adaptation [56]. Recent work [57] demonstrated that placental overexpression of PAPPA2 strongly predicts the most severe forms of preeclampsia (PE), particularly among individuals without preexisting risk factors, and that its expression correlates with an earlier gestational age at delivery in a dose-dependent manner. However, this study did not evaluate whether PAPPA2 is specific to PE or whether it reflects a broader risk signal for preterm labor and delivery. Our findings suggest a complementary and potentially broader role: PAPPA2 is significantly elevated in maternal serum of individuals who deliver preterm even in the absence of clinical PE, supporting its utility as a biomarker of sPTB. The dose-dependent relationship observed in placental tissue may, in fact, reflect underlying mechanisms of early placental dysfunction that precede both sPTB and PE. Thus, PAPPA2 may serve as an early marker of impaired placental adaptation across a spectrum of pregnancy complications, not limited to PE.

NID1, a component of the extracellular matrix, may influence cell adhesion at the maternal–fetal interface [58], while GPX3, an extracellular antioxidant enzyme, protects against oxidative damage and has been implicated in inflammatory pathways known to trigger preterm labor [59]. These three proteins converge on biologically relevant axes, placental invasion, oxidative stress, and extracellular remodeling—that are implicated in the pathophysiology of sPTB.

Beyond biological relevance and predictive performance, our model offers practical advantages. It was validated using serum samples from multiple time points, allowing for flexible sampling windows throughout gestation. It also utilizes only three input features, thereby reducing overfitting and facilitating a cost-effective implementation. We confirmed a strong correlation between the LC–MS/MS and ELISA platforms for all three proteins, supporting the potential deployment in clinical laboratories without reliance on mass spectrometry infrastructure.

Subgroup analyses distinguishing preterm prelabor rupture of membranes (PPROM +) from spontaneous preterm labor with intact membranes (PPROM −) demonstrated that the three-protein panel performed comparably across these phenotypes (Fig. 7A, B). This consistency indicates that the biomarker signal reflects shared upstream biological pathways, including oxidative stress, extracellular matrix remodeling, and impaired trophoblast–decidual interactions, rather than processes unique to membrane rupture. Importantly, all samples were collected before the onset of labor or membrane rupture, confirming that the observed associations are predictive rather than reactive. Comparative analyses further showed that the three-protein panel consistently outperformed the IBP4/SHBG ratio across both PPROM + and PPROM − subgroups and at both < 34- and < 37-week thresholds (Fig. 7A, B). Whereas the IBP4/SHBG ratio primarily reflects late-gestation hormonal and metabolic changes, the three-protein signature appears to capture earlier, upstream placental and extracellular-matrix processes common to both spontaneous labor and membrane-rupture etiologies. The superior and more stable performance of the three-protein panel, particularly in early gestation, supports its value as a biologically grounded and clinically actionable predictor of spontaneous preterm birth across diverse etiologic subtypes. Our analyses incorporated PPROM + cases regardless of subsequent labor induction, consistent with standard clinical classification of spontaneous preterm birth following prelabor membrane rupture. This ensured that biomarker performance reflected true spontaneous etiologies rather than provider-initiated deliveries.

The timing of biomarker-based risk assessment is clinically critical, as distinct gestational windows inform different types of interventions. Our trimester-stratified analysis (Table 6) demonstrated that the three-protein panel retains strong predictive accuracy across the first and second trimesters, the period most relevant for preventive interventions such as low-dose aspirin and progesterone therapy, both recommended for initiation before 16–20 weeks’ gestation [60]. Early identification of high-risk pregnancies within this window enables proactive care before irreversible placental maladaptation occurs. Beyond early pregnancy, maintaining predictive performance into the third trimester provides additional clinical utility—supporting individualized decisions regarding antenatal corticosteroids, magnesium sulfate administration, and in utero transfer for neonatal care optimization [13, 61]. Thus, evaluation across gestational windows reflects not only biological robustness but also the diverse clinical contexts in which a preterm-birth risk test would be deployed.

Ethnic and regional differences in biomarker performance were evident in this study, with the IBP4/SHBG ratio showing reduced predictive accuracy in the Asian cohorts compared to the two different region US cohorts. This observation is consistent with findings in sub-Saharan African women, where the same ratio demonstrated lower predictive value (*AUC*≈0.64) relative to the populations for which it was originally developed [20]. The underlying mechanisms likely reflect complex interactions among genetic, metabolic, and environmental factors. Although a recent cross-ancestry genome-wide association study found no evidence of ancestry-specific genetic effects for circulating SHBG, estradiol, or testosterone concentrations [62], metabolic and hormonal regulation involving SHBG and IGFBP4 may differ across populations due to variation in adiposity, diet, endocrine milieu, and placental function. Previous studies have also shown that the association between SHBG and adiposity differs by race [63], suggesting context-dependent metabolic signaling that may influence biomarker performance.

Regarding IBP4, available genomic data show that while several loci influence IBP4 expression and circulating protein levels, no evidence of ancestry-specific effects has been reported [64, 65]. However, population differences in IGF-axis regulation and placental IBP4 methylation patterns [66] and metabolic factors such as adiposity [66] have been described, which may contribute to differential predictive performance across ethnicities. These observations support the interpretation that the IBP4/SHBG ratio’s reduced predictive strength in Asian and sub-Saharan African cohorts likely arises from contextual physiological and environmental heterogeneity, not from ancestry-specific genetic divergence.

In contrast, current genetic evidence does not indicate strong ancestry-specific effects for the three-protein panel components, GPX3, NID1, and PAPPA2. GWAS analyses have implicated NID1 in nevus count [67] and diabetic kidney disease [67] without reporting ancestry-linked heterogeneity; GPX3 resides in a cross-ancestry locus (GPX3–TNIP1) associated with amyotrophic lateral sclerosis [68] and Alzheimer’s disease [68] with consistent effects across populations; and PAPPA2 has been associated with birth weight [69, 70] and elevated expression in preeclampsia [56, 71] but not with ancestry-specific genetic variation. Large-scale pregnancy GWAS on gestational duration and preterm birth likewise have not identified ancestry-specific signals for these proteins [70]. Furthermore, emerging multi-ancestry proteogenomic resources indicate that many circulating proteins, including GPX3, NID1, and PAPPA2, exhibit substantial cross-ancestry sharing of pQTL architecture (with some ancestry-specific signals), as shown in large UK Biobank analyses [72, 73]. Collectively, these findings suggest that the three-protein panel may be less sensitive to ancestry-related genetic differences than ratios such as IBP4/SHBG. Nonetheless, future cross-ancestry pQTL and colocalization analyses in pregnancy cohorts will be essential to confirm generalizability and ensure equitable clinical performance.

Collectively, these findings demonstrate that the three-protein panel performs robustly across pathophysiologic subtypes and gestational thresholds, outperforming existing mid-gestation markers and maintaining predictive value early in pregnancy. While these results highlight the panel’s translational promise, several methodological considerations merit discussion. First, our evaluation employed a nested case–control design within prospective longitudinal cohorts, which inherently enriched the analytic datasets with sPTB cases and increased case–control ratios relative to true population prevalence. This approach allowed unbiased pre-outcome biomarker assessment and efficient use of biospecimens, but it limits direct estimation of population-level predictive values. We mitigated potential spectrum effects by reporting window-restricted performance, prevalence-weighted PPV and NPV estimates, and cross-cohort validation across diverse clinical and geographic settings. While the model maintained robust performance after prevalence adjustment, prospective, population-level validation remains essential to confirm real-world predictive accuracy. A full-cohort validation using the registered NCT06664554 study is underway to address this next step. Second, although our cohorts included both US and Asian populations, sample-collection frameworks differed—Stanford and UAB used serial longitudinal sampling across all trimesters, whereas Asian cohorts contributed primarily single time-point collections during routine antenatal visits. These healthcare-system differences explain variations in sampling density but also enhance the generalizability of findings across heterogeneous clinical environments. Third, the number of available samples in the earliest gestational window (≤ 13 weeks) was limited, particularly in the US cohorts, which may affect the precision of early-trimester estimates. Data from the NCT06664554 cohort provides additional early-pregnancy samples to strengthen calibration in this window. Fourth, all analyses were restricted to singleton pregnancies without major fetal anomalies. Although this minimizes biological heterogeneity, it limits immediate extrapolation to multifetal gestations, which carry distinct mechanistic risks for preterm birth. Finally, the present work used standardized targeted-proteomics assays; while analytically robust, translation into clinical-grade immunoassays or high-throughput platforms will require harmonization of calibration standards and inter-laboratory reproducibility testing. Addressing these aspects through multicenter analytical validation and implementation studies will be essential before routine clinical adoption.

## Conclusion

This study provides a robust, multi-cohort validation of a biologically informed and technically scalable three-protein serum biomarker panel (GPX3, NID1, and PAPPA2) for early risk assessment of spontaneous preterm birth (sPTB). The panel demonstrated reproducible performance across geographically and ethnically diverse populations, retained accuracy in both PPROM + and PPROM − phenotypes, and achieved its strongest discrimination in the first and second trimesters, when preventive therapies such as progesterone, cerclage, and low-dose aspirin are most effective. Compared to existing mid-gestation tools, such as the IBP4/SHBG ratio, this panel showed superior performance and is compatible with standard clinical laboratory platforms. These findings support the clinical potential of this biomarker signature as an early, population-deployable screening tool for sPTB. Future work should focus on integrating the test into prenatal care workflows and elucidating the mechanistic roles of PAPPA2, NID1, and GPX3 in the timing of parturition, to enable more effective prevention of preterm birth globally.

## Supplementary Information

Supplementary Material 1.

Supplementary Material 2.

Supplementary Material 3.

Supplementary Material 4.

## Data Availability

De-identified datasets and software code used in this study will be made available in a GitHub repository upon publication ([https://github.com/bxlinglaboratory/Preterm-Birth-Protein-Predictor](https:/github.com/bxlinglaboratory/Preterm-Birth-Protein-Predictor)). The LC–MS/MS raw proteomics datasets generated and/or analyzed during the current study are available in the PeptideAtlas repository under accession PASS05936 ([https://db.systemsbiology.net/sbeams/cgi/PeptideAtlas/PASS\_View?identifier = PASS05936](https:/db.systemsbiology.net/sbeams/cgi/PeptideAtlas/PASS_View?identifier = PASS05936)).

## Abbreviations

sPTB: Spontaneous preterm birth
PTB: Preterm birth
PPROM: Preterm premature rupture of membranes
GA: Gestational age
BMI: Body mass index
LMP: Last menstrual period
IVF: In vitro fertilization
LC–MS/MS: Liquid chromatography–tandem mass spectrometry
ELISA: Enzyme-linked immunosorbent assay
AUC: Area under the receiver operating characteristic curve
ROC: Receiver operating characteristic
CI: Confidence interval
OR: Odds ratio
SD: Standard deviation
IQR: Interquartile range
FDR: False discovery rate
DEG: Differentially expressed gene
GPX3: Glutathione peroxidase 3
NID1: Nidogen 1
PAPPA2: Pregnancy-associated plasma protein A2
IBP4: Insulin-like growth factor-binding protein 4
SHBG: Sex hormone-binding globulin
IRB: Institutional Review Board
