# Supplementary material for: Early gestational prediction of spontaneous preterm birth using a validated three-protein serum biomarker panel
Source: BMC Med. 2026 Feb 2;24:138. doi: 10.1186/s12916-026-04639-9 (PMC12955166; doi:10.1186/s12916-026-04639-9)
Supplement: Supplementary file 1 — Supplementary Material 1. [file 12916_2026_4639_MOESM1_ESM.pdf]

	Symbol	fold	pval	threshold
1	AASDHPPT	1.23544466	0.00762308	Up
2	AASS	1.24631964	0.01863161	Up
3	ABCA1	1.26028321	0.01606398	Up
4	ABCA8	1.45385129	0.00301036	Up
5	ABCB1	1.58621847	2.7833E-05	Up
6	ABI3BP	1.33300854	0.0013988	Up
7	ACE2	1.23081738	0.03427121	Up
8	ACKR2	0.82632229	0.01451512	Down
9	ACOXL	0.75618523	0.00643698	Down
10	ACSL1	1.42584201	0.01221742	Up
11	ACSL3	1.22925532	0.00869366	Up
12	ACTA2	1.63980909	0.01037032	Up
13	ACTB	1.51727765	0.00010352	Up
14	ACTG2	2.00347305	0.00043474	Up
15	ACTN1	1.4858002	0.00025667	Up
16	ACTR2	1.5118953	0.00035813	Up
17	ADAM19	1.2155315	0.01819686	Up
18	ADAM28	1.48658891	0.00094486	Up
19	ADAM9	1.49239741	6.7055E-05	Up
20	ADAMTS1	1.58907367	4.5131E-05	Up
21	ADAMTS2	1.45198268	0.0005364	Up
22	ADAMTS5	2.23244615	4.7111E-07	Up
23	ADAMTS9	1.30203756	2.2788E-05	Up
24	ADARB1	1.41402472	0.00435828	Up
25	ADD3	1.21667867	0.01299571	Up
26	ADIRF	0.76495486	0.01729366	Down
27	AFF1	1.25486598	0.00071715	Up
28	AFF3	0.6877656	0.00040779	Down
29	AGA	1.20166885	0.03328472	Up
30	AGL	1.21006956	0.00402625	Up
31	AGPAT4	1.30085801	0.00077396	Up
32	AGPAT5	1.39198098	0.00051941	Up
33	AHNAK2	1.48057147	1.9629E-05	Up
34	AHR	0.80620042	0.01861764	Down
35	AKAP13	1.5926054	0.01032474	Up
36	ALCAM	1.25517312	0.00820415	Up
37	ALDH1A1	1.86718833	1.6294E-05	Up
38	ALDH1B1	1.24479808	0.00209132	Up
39	ALDH2	1.24431931	0.01317684	Up
40	ALDH3B2	0.7221473	0.00067086	Down
41	ALDH6A1	1.23012888	0.03151493	Up
42	ALOX5AP	1.24434572	0.0004184	Up
43	ALPP	0.59785531	0.00010656	Down
44	AMD1	0.77544968	0.00115978	Down
45	ANGPTL2	1.34437172	0.00650379	Up
46	ANK2	1.34998482	0.01016252	Up
47	ANKRD1	1.23609331	0.00164533	Up
48	ANO2	0.81408152	0.00769501	Down
49	ANP32A	1.22413449	0.005753	Up
50	ANP32E	1.35408043	0.00019286	Up
51	ANPEP	1.44629222	0.00012791	Up

52	ANTXR1	1.43137735	0.00196531	Up
53	ANXA1	1.9369352	1.6883E-05	Up
54	ANXA3	1.21492447	0.01192648	Up
55	AOC1	1.29929996	0.00022824	Up
56	AOC3	1.38042326	0.02515762	Up
57	AP2B1	1.22318616	0.00795885	Up
58	APOBEC3C	1.21427006	0.00953421	Up
59	APOD	1.58918281	0.00010613	Up
60	APOE	1.51340427	0.00119838	Up
61	APOL6	1.30233566	0.02220731	Up
62	APOLD1	1.62272967	0.0003233	Up
63	APP	1.20186009	0.03827159	Up
64	AREG	1.21404239	7.6195E-05	Up
65	ARFIP1	1.2016028	0.00404764	Up
66	ARG2	1.21047081	0.04481755	Up
67	ARHGAP17	0.83001562	0.02428809	Down
68	ARHGAP24	1.2115513	0.01300073	Up
69	ARHGAP28	1.43459739	0.00038468	Up
70	ARHGAP6	1.25416308	0.01983327	Up
71	ARHGEF12	0.82685089	0.01463718	Down
72	ARHGEF7	1.46670776	0.00029024	Up
73	ARL3	1.20542564	0.02698933	Up
74	ARL4C	0.76115613	0.00360484	Down
75	ARPC2	1.28002271	0.01130792	Up
76	ARSJ	1.21760049	0.03675899	Up
77	ART4	1.33183886	0.04698899	Up
78	ARTN	0.7593872	0.00154482	Down
79	ASAP3	1.32862164	1.2018E-05	Up
80	ASB4	2.19856673	6.7453E-05	Up
81	ASCL2	1.4260852	0.00028523	Up
82	ASPA	1.25530098	0.00466953	Up
83	ASPN	1.34701847	0.02307813	Up
84	ASS1	1.24901479	0.00551546	Up
85	ASXL1	1.21683009	0.04538825	Up
86	ATF1	0.79596106	0.02893011	Down
87	ATF2	1.25695628	0.01954092	Up
88	ATM	1.32863079	0.00060427	Up
89	ATP13A3	1.44773577	6.2064E-05	Up
90	ATP1A1	0.83080857	0.0360903	Down
91	ATP1B1	1.67601241	2.1987E-05	Up
92	ATP1B3	0.82666156	0.01138536	Down
93	ATP2B1	1.37564717	0.00036614	Up
94	ATP2B4	1.28319866	0.00466953	Up
95	ATP6V0A4	1.49229377	0.00945797	Up
96	ATP8A2	1.37437381	0.01683551	Up
97	ATP8B4	1.35670572	0.00360484	Up
98	ATXN1	1.35433088	0.01210928	Up
99	ATXN3	1.25702407	0.00315388	Up
100	ATXN7	0.76523625	0.00390267	Down
101	ATXN7L3B	1.35639964	0.00068392	Up
102	AVIL	0.83121548	0.01156123	Down
103	AXL	1.31953916	0.0086507	Up

104	B4GALT1	0.76111078	0.018912	Down
105	BAG2	1.50345754	2.161E-05	Up
106	BAMBI	1.5303574	1.4172E-05	Up
107	BARD1	1.25537378	0.00980444	Up
108	BCAT1	1.20978995	0.00488783	Up
109	BCL2	0.77242417	0.03695568	Down
110	BCL6	1.41220997	3.3775E-05	Up
111	BCLAF1	1.65306132	7.2735E-05	Up
112	BHLHE40	1.24923858	2.6277E-07	Up
113	BHLHE41	1.2851442	0.01389528	Up
114	BICC1	1.35377888	0.00474081	Up
115	BIN2	1.81366227	2.1427E-05	Up
116	BMP1	0.73497342	0.00167349	Down
117	BMP2	1.41643536	0.00619485	Up
118	BMP5	1.38106058	0.00151599	Up
119	BMP7	1.53528872	0.00014393	Up
120	BNC2	1.45040895	7.1341E-05	Up
121	BNIP3	1.27959915	0.02074114	Up
122	C1orf21	1.22244876	0.01884728	Up
123	C1QB	1.29535313	0.00217996	Up
124	C1R	1.5228917	0.00050507	Up
125	C1RL	1.20746063	0.01005117	Up
126	C1S	1.31231053	0.00196952	Up
127	C2	1.28708502	0.00176768	Up
128	C3	1.45292878	0.00022587	Up
129	C4orf19	0.83207409	0.0299587	Down
130	C6orf62	1.30548521	0.00033281	Up
131	CA10	0.71715036	0.00377265	Down
132	CA12	1.27844185	0.00253029	Up
133	CACNG4	0.81161902	0.04596499	Down
134	CADM3	1.31756219	0.00723083	Up
135	CALCRL	1.23625402	0.03819394	Up
136	CALD1	2.3815587	0.00091821	Up
137	CAMK2G	0.7467705	0.0034038	Down
138	CAMK2N1	1.20178596	0.0212839	Up
139	CANT1	0.81566402	0.00888014	Down
140	CAPG	1.37659472	0.00021566	Up
141	CAPN6	0.76138635	0.01883716	Down
142	CAPRIN1	0.80987647	0.04437801	Down
143	CAPRIN2	1.2273172	0.01009207	Up
144	CASK	1.23016174	0.00036021	Up
145	CAT	1.20214745	0.00040206	Up
146	CBX3	1.26458865	0.00291991	Up
147	CBX5	1.36957491	0.00140137	Up
148	CCL4	1.26002976	0.0107533	Up
149	CCND1	0.80642172	0.02103482	Down
150	CCND2	1.57302694	4.7605E-06	Up
151	CCNE1	1.34952707	2.8559E-05	Up
152	CCNG2	1.42752497	3.2641E-05	Up
153	CCP110	1.37810336	0.00143061	Up
154	CCR1	1.30310109	0.00426693	Up
155	CD163	1.62257108	1.1256E-05	Up

156	CD200	0.78930497	0.00079234	Down
157	CD248	1.74657317	3.0094E-06	Up
158	CD320	0.76831628	0.00448531	Down
159	CD36	1.43626335	0.04878254	Up
160	CD44	1.2004187	0.0014734	Up
161	CD53	1.22208079	0.00492781	Up
162	CD86	1.20853708	6.1228E-05	Up
163	CD9	1.46843722	1.8776E-05	Up
164	CDC14B	1.27796686	0.00029634	Up
165	CDC42	1.36204107	0.00333105	Up
166	CDCP1	1.21210228	0.03738211	Up
167	CDH11	1.69986599	0.00031215	Up
168	CDH3	1.27578839	0.03461577	Up
169	CDK2	1.23632029	0.01420465	Up
170	CDKN1A	0.7488751	0.00031682	Down
171	CDKN1C	1.37129967	0.03413412	Up
172	CDS1	0.79395173	0.03653863	Down
173	CDV3	1.29757851	0.0069781	Up
174	CEACAM1	0.83133902	0.00065947	Down
175	CELF2	1.25809478	0.0124786	Up
176	CENPT	0.81853683	0.00102306	Down
177	CERS6	1.45760943	0.00037053	Up
178	CETP	0.81760757	0.01121653	Down
179	CFD	1.32525548	0.00498606	Up
180	CFDP1	1.56363	8.3895E-05	Up
181	CFH	1.2705432	0.00139873	Up
182	CFLAR	1.24943373	0.00043832	Up
183	CGA	1.31841334	0.0005841	Up
184	CHD9	1.37891791	0.01243155	Up
185	CHI3L2	1.48102804	0.00125774	Up
186	CHIC2	1.20490751	0.02706915	Up
187	CHN2	1.24497021	0.01916628	Up
188	CHPT1	1.20693789	0.00023573	Up
189	CHST2	1.49428879	0.0002733	Up
190	CILP	1.45338609	0.00423289	Up
191	CITED2	1.89277399	1.5154E-06	Up
192	CKB	1.22682517	0.02880535	Up
193	CLDN1	0.65873534	0.00024011	Down
194	CLDN8	0.80435614	0.00086964	Down
195	CLIC4	1.47490338	0.00078596	Up
196	CLIP4	0.7862155	0.01537707	Down
197	CLMN	0.82923585	4.453E-06	Down
198	CLSTN2	1.3850652	0.02592872	Up
199	CLU	1.22214124	0.00042464	Up
200	CMAHP	0.77195275	0.0024449	Down
201	CMAS	1.31527671	0.01028175	Up
202	CMKLR1	1.23154855	0.00510762	Up
203	CNGA1	0.76467923	0.00154139	Down
204	CNN1	1.41170117	0.02396163	Up
205	CNN2	1.33724258	0.00739788	Up
206	CNR1	1.91943648	2.1551E-05	Up
207	COASY	0.7776865	0.01298677	Down

208	COL11A1	1.42661927	0.00056582	Up
209	COL11A2	0.81608787	0.01001492	Down
210	COL14A1	1.33828311	0.00011311	Up
211	COL1A1	1.47019747	0.00056377	Up
212	COL1A2	1.72737887	5.9171E-05	Up
213	COL21A1	1.33576735	0.02962788	Up
214	COL3A1	1.63183021	0.0002389	Up
215	COL4A2	1.21602678	0.00393063	Up
216	COL4A4	1.22195342	0.03112881	Up
217	COL4A6	1.33491547	0.00621031	Up
218	COL5A1	1.42189629	0.00708021	Up
219	COL5A2	1.33750965	0.00122388	Up
220	COL6A1	1.41127161	0.00039112	Up
221	COL6A2	1.49487836	0.00106909	Up
222	COL6A3	1.80393757	4.0951E-06	Up
223	COPA	1.3116998	0.00522817	Up
224	COPS8	1.2123892	0.02752571	Up
225	COQ2	1.26104371	0.01799688	Up
226	COTL1	1.29288978	0.00274064	Up
227	CPM	1.42420538	0.01275946	Up
228	CPS1	2.77900094	5.88E-08	Up
229	CR1	1.25468938	0.00051578	Up
230	CRABP2	1.40903336	0.00619533	Up
231	CRCT1	1.53955502	0.03352393	Up
232	CREB1	1.30684584	0.00157011	Up
233	CREB3L1	1.28271157	0.00714328	Up
234	CRH	0.56030378	2.7758E-05	Down
235	CRHBP	1.63197737	0.00270752	Up
236	CRIP1	0.76592466	0.00657084	Down
237	CRIP2	0.71844346	8.4422E-05	Down
238	CRISPLD2	1.54939247	0.00179291	Up
239	CRLF1	1.46430824	0.00397886	Up
240	CSF3R	0.7574957	0.01032474	Down
241	CSNK1A1	1.24201107	0.02118602	Up
242	CSRP2	1.28190286	0.03602713	Up
243	CSTA	1.25467155	0.00060799	Up
244	CSTB	1.2408626	0.04074278	Up
245	CTNNB1	1.3628048	0.0060552	Up
246	CTSB	1.33392188	0.00151476	Up
247	CTSC	1.30485444	0.00024335	Up
248	CTSW	1.43291914	0.00587375	Up
249	CUL4A	1.24765749	0.00271555	Up
250	CUX1	1.24702867	0.0211146	Up
251	CX3CL1	0.76515417	0.00389109	Down
252	CXCL1	1.66912253	0.01115164	Up
253	CXCL14	1.81015658	4.4121E-06	Up
254	CYBRD1	1.2399177	0.00113092	Up
255	CYFIP2	1.22590674	0.03883216	Up
256	CYP24A1	1.54506237	0.03232934	Up
257	CYSLTR2	1.23330668	0.00220745	Up
258	DAAM1	1.32375924	0.02409214	Up
259	DAPK1	0.73971542	1.2343E-05	Down

260	DAPP1	1.3059975	0.00041634	Up
261	DBI	1.23592126	0.00903292	Up
262	DCN	1.53566055	9.6194E-05	Up
263	DCX	0.81580019	0.01335956	Down
264	DDAH1	1.51550018	0.00026602	Up
265	DDIT4	1.44276952	0.00767699	Up
266	DDX17	2.06314586	1.4387E-05	Up
267	DDX3X	1.44537481	0.0011554	Up
268	DECR1	1.26009465	0.0282502	Up
269	DENND2A	1.24272962	0.02760613	Up
270	DEPTOR	1.52034166	8.386E-05	Up
271	DES	1.46074772	0.01696102	Up
272	DGKH	1.40012844	0.01500523	Up
273	DHRS2	1.27419181	1.4604E-05	Up
274	DHRS9	1.26096926	0.0361385	Up
275	DHX9	1.21022807	0.00487579	Up
276	DIO2	1.61077012	3.9955E-06	Up
277	DIO3	1.51931417	0.00241245	Up
278	DKK1	2.73296452	2.5484E-07	Up
279	DLEU2	1.2587432	0.0054016	Up
280	DLGAP1	1.27672722	0.00847563	Up
281	DNAJB14	1.23454627	0.00781059	Up
282	DNAJB4	1.33580789	0.01495925	Up
283	DNAJC15	1.2543855	0.01611124	Up
284	DNAJC3	1.63832608	0.00055883	Up
285	DNMT3B	1.28681074	0.01022638	Up
286	DOCK5	1.21516204	0.00515091	Up
287	DPP4	1.27412472	0.00032742	Up
288	DPT	1.46453442	0.00031682	Up
289	DPY19L1	1.21501832	0.00019742	Up
290	DSC2	1.20445721	0.01234746	Up
291	DST	1.4345375	0.00055883	Up
292	DTNB	0.79361763	0.00987004	Down
293	DVL3	0.80564155	0.01611403	Down
294	DYNC1H1	1.28191353	0.01289783	Up
295	EBI3	0.80646438	0.00046216	Down
296	EDA2R	0.73155521	0.00195776	Down
297	EDIL3	1.36068707	0.03990809	Up
298	EDNRA	1.38354215	0.03232934	Up
299	EDNRB	1.36454701	0.02410002	Up
300	EFCAB2	0.82380918	0.01077182	Down
301	EFEMP1	1.35139994	6.3339E-06	Up
302	EFHD1	0.79462748	0.01927484	Down
303	EFNA5	1.40092976	0.01063483	Up
304	EFNB2	1.21083429	0.00760516	Up
305	EFS	0.8310816	0.02474677	Down
306	EGFL6	1.36206251	0.0034922	Up
307	EGLN3	1.44060765	0.00061017	Up
308	EGR1	1.26509195	7.8375E-05	Up
309	EHBP1	1.46063212	0.00111949	Up
310	EIF1AX	1.24616952	0.03745863	Up
311	EIF1AY	1.23357577	0.00037611	Up

312	EIF3B	1.31742866	0.00336705	Up
313	EIF4EBP1	1.29146148	0.02061319	Up
314	ELL2	1.34581711	0.00602585	Up
315	ELOVL2	1.68892313	5.24E-07	Up
316	EMILIN2	1.38278485	0.02128698	Up
317	EMP2	0.7888304	0.00478958	Down
318	EMP3	1.28846309	0.01977925	Up
319	ENAH	1.36167682	7.5203E-05	Up
320	ENDOU	0.7763134	0.01141626	Down
321	ENO1	1.36643292	0.00019562	Up
322	ENPEP	1.26719421	0.00045206	Up
323	ENPP1	1.44233173	4.4883E-06	Up
324	ENPP2	1.29961286	6.8007E-05	Up
325	EPAS1	1.24861844	0.00159499	Up
326	EPHB6	0.81261397	0.00230331	Down
327	EPOR	1.20482804	0.0111862	Up
328	EPS8	1.30910621	0.01009668	Up
329	ERAP2	1.51751534	0.00958781	Up
330	ESRRG	1.24382504	1.4835E-05	Up
331	ETS2	1.24107339	0.00109	Up
332	EVI2A	1.20306543	0.03930799	Up
333	EXPH5	0.67652475	0.00392994	Down
334	EXTL3	1.37279125	0.00010634	Up
335	EZR	1.33183188	0.00039474	Up
336	F2R	1.47540795	3.3066E-05	Up
337	F3	1.34907573	0.00026026	Up
338	F5	1.25691714	0.00011968	Up
339	FABP4	0.56877772	4.4253E-05	Down
340	FADS2	1.28498287	0.01848574	Up
341	FAM107A	1.31583565	2.1096E-05	Up
342	FAM110B	1.3430442	0.00587883	Up
343	FAM184A	1.2778945	0.00913701	Up
344	FAP	1.358273	0.00191932	Up
345	FAR2	1.21362269	0.01196608	Up
346	FARP1	1.23457303	0.01754354	Up
347	FAT2	1.3321437	0.00611321	Up
348	FBLN1	1.34506553	0.01420104	Up
349	FBLN5	1.54453789	0.00011786	Up
350	FBN1	1.41855335	2.8006E-05	Up
351	FBN2	0.74311977	0.00040097	Down
352	FBXL5	1.27646394	0.00610242	Up
353	FCER1G	1.27934214	0.00440651	Up
354	FCGBP	1.21756025	0.01265052	Up
355	FERMT2	1.44141045	0.0094873	Up
356	FGF7	1.27874908	0.0160269	Up
357	FGFR1	1.31023588	0.0005984	Up
358	FGL1	1.40396428	0.0292616	Up
359	FGL2	1.25120085	0.00221003	Up
360	FHOD3	1.20977719	0.02091373	Up
361	FILIP1L	1.37640641	0.00057779	Up
362	FKBP11	1.2873942	0.02537052	Up
363	FKBP14	1.2239849	0.0056371	Up

364	FKBP5	1.56395579	1.0573E-05	Up
365	FLNB	1.39720821	0.00069554	Up
366	FLRT3	1.28517464	0.04893936	Up
367	FLT1	1.34162457	2.6234E-05	Up
368	FLT4	0.74039203	5.5934E-05	Down
369	FN1	1.89356747	4.9011E-05	Up
370	FOLR1	1.40767876	0.00013787	Up
371	FOS	1.22403853	0.00102225	Up
372	FOSL2	1.37763505	0.0015214	Up
373	FOXJ3	0.79121454	0.01722087	Down
374	FRMD4A	1.27773178	0.0012935	Up
375	FST	1.29225519	0.04228129	Up
376	FSTL3	1.82440908	0.00042012	Up
377	FTH1	1.42845598	0.00069583	Up
378	FTL	1.23432536	0.00124912	Up
379	FTSJ1	1.20404381	0.00485203	Up
380	FURIN	0.79053389	0.0394902	Down
381	FUS	1.5371113	0.0001086	Up
382	FUT9	1.2934045	0.00638938	Up
383	FXR1	1.25157016	0.01491521	Up
384	FYN	1.27510214	0.0044417	Up
385	FZD1	0.77563916	0.00705161	Down
386	FZD7	0.80994811	6.3717E-06	Down
387	G3BP1	1.28736939	0.00636943	Up
388	G3BP2	1.27166691	0.01067244	Up
389	GABRA4	0.7612145	0.00060624	Down
390	GABRR2	0.82328338	0.01900544	Down
391	GADD45A	1.32783676	0.00064384	Up
392	GALNT1	1.2071748	0.00404885	Up
393	GALNT3	1.38228963	1.5032E-05	Up
394	GALNT6	1.38727997	0.00054288	Up
395	GALNT7	1.24371762	0.00137042	Up
396	GAP43	1.63233036	0.00233963	Up
397	GAS1	1.85848063	0.0003233	Up
398	GATA4	1.21352856	0.01034042	Up
399	GATA6	1.49947747	0.00035264	Up
400	GATM	1.35863867	2.221E-05	Up
401	GBP2	0.79408818	0.00197582	Down
402	GCNT1	1.21791254	0.00126541	Up
403	GDAP2	1.27192955	0.00216203	Up
404	GDI2	1.27286807	0.00505165	Up
405	GDPD5	0.78871436	0.02798177	Down
406	GFOD2	0.79396651	0.00178267	Down
407	GGCX	1.33136383	0.01718782	Up
408	GGH	1.36274067	0.03033401	Up
409	GGT5	1.3438992	0.04275249	Up
410	GH2	0.75707573	0.03141279	Down
411	GJA1	1.3859296	0.00508054	Up
412	GJC1	1.80112104	0.00011969	Up
413	GLIPR1	1.68179064	1.9767E-06	Up
414	GLO1	1.23147632	0.00323695	Up
415	GLS	1.28766507	0.00129917	Up

416	GLUL	1.38389532	0.00202066	Up
417	GNA12	1.25801818	0.00044811	Up
418	GNA13	1.20106992	0.00775618	Up
419	GNB5	1.223743	0.02019907	Up
420	GNGT1	0.82970525	0.01464785	Down
421	GNL3L	1.23377601	0.00859174	Up
422	GNLY	2.04001247	1.7657E-05	Up
423	GNPTAB	1.25488218	0.00852451	Up
424	GOLIM4	1.27435548	0.02537052	Up
425	GPNMB	0.7867438	0.00121865	Down
426	GPR137B	1.22765624	0.01225994	Up
427	GPRC5A	1.30346384	0.00121938	Up
428	GPRC5B	1.21756734	0.04095161	Up
429	GPX1	1.24973013	0.00146192	Up
430	GPX3	1.65997048	0.00043987	Up
431	GRB14	0.83234775	0.013217	Down
432	GRB7	0.8229427	0.01527751	Down
433	GREM2	1.59300296	7.2354E-05	Up
434	GSTM3	1.37862086	0.00096019	Up
435	GTF2H5	1.21185549	0.00068365	Up
436	GYG2	1.20842758	0.04167827	Up
437	GYS1	1.257677	0.01060604	Up
438	HAND2	1.29139589	0.02109785	Up
439	HAPLN1	1.86571594	1.3131E-05	Up
440	HBB	0.63576035	2.7572E-06	Down
441	HBD	0.75898162	4.5573E-08	Down
442	HCAR3	1.34099154	0.0208344	Up
443	HDAC5	0.81475256	0.02312775	Down
444	HES1	0.81176801	0.00224733	Down
445	HEY2	1.20822313	0.03906884	Up
446	HGF	1.55874483	1.0316E-07	Up
447	HIF3A	1.88494645	0.0017549	Up
448	HINT1	1.24848179	0.04152533	Up
449	HIPK2	1.33534138	0.01598392	Up
450	HIPK3	1.50061086	0.00018422	Up
451	HK2	1.46419211	0.00826284	Up
452	HLA-DQA1	1.21526983	0.00574786	Up
453	HLF	0.74833179	0.00565273	Down
454	HLTF	1.233475	0.00041511	Up
455	HMGB2	1.23273244	0.04413269	Up
456	HMGCS1	1.26921837	0.00579428	Up
457	HMOX1	1.26238911	8.4317E-05	Up
458	HNMT	1.32185795	0.00046183	Up
459	HNRNPH1	1.20677851	0.00640674	Up
460	HOMER1	1.34772108	0.02632642	Up
461	HOXA10	1.24020445	0.02347406	Up
462	HOXC6	1.25565287	0.03010565	Up
463	HP1BP3	0.79879226	0.0028925	Down
464	HPGDS	1.30626809	0.00378091	Up
465	HPRT1	1.41579634	0.0097111	Up
466	HSD11B1	1.21825922	0.00068807	Up
467	HSD17B1	0.79539247	0.02100838	Down

468	HSD17B11	1.29308039	0.00929959	Up
469	HSD17B6	1.26379514	0.00597759	Up
470	HSD3B1	1.59712609	4.4024E-05	Up
471	HSP90AA1	1.22088325	0.01063912	Up
472	HSP90AB1	1.48364192	0.00044629	Up
473	HSPA2	1.37299468	0.02428586	Up
474	HSPB6	1.3679396	0.00112576	Up
475	HSPD1	1.46280288	0.00070563	Up
476	HSPE1	1.20212237	0.04648094	Up
477	HSPG2	1.3746555	0.00028263	Up
478	HSPH1	1.31747089	0.0010302	Up
479	HTR2B	1.39910354	0.00536853	Up
480	HUNK	1.25829089	0.0006616	Up
481	ID4	1.95506892	0.00051452	Up
482	IDH1	1.27370273	0.03499261	Up
483	IFI16	1.52557509	6.9882E-06	Up
484	IFI6	1.2931268	0.00324908	Up
485	IFITM3	1.21692961	0.0007044	Up
486	IFNGR2	1.28855603	0.00847089	Up
487	IGF1	1.52595255	1.9566E-05	Up
488	IGF1R	1.22266808	0.02354001	Up
489	IGF2BP3	1.82164894	1.9578E-05	Up
490	IGFBP2	1.37981979	0.00039348	Up
491	IGFBP3	1.27798888	0.00820115	Up
492	IGFBP5	1.50658595	0.00052768	Up
493	IGFBP7	1.28671347	0.00416704	Up
494	IGKC	3.07142466	7.2735E-05	Up
495	IL10RA	1.20711468	0.00045382	Up
496	IL13RA1	1.20760742	0.00375789	Up
497	IL18R1	1.33065594	0.01092725	Up
498	IL1B	1.27765453	0.00044675	Up
499	IL1R2	2.41739477	9.2736E-10	Up
500	IL2RB	1.58095343	2.238E-05	Up
501	IL6ST	2.76203474	3.7147E-06	Up
502	IQGAP1	1.54807661	0.00022945	Up
503	IRAK3	1.33014193	0.02980133	Up
504	IRS1	1.62555158	7.4816E-05	Up
505	ISG15	1.36636972	0.00764102	Up
506	ISLR	1.21566995	0.00109504	Up
507	ITGA9	1.22034918	0.00829321	Up
508	ITGAM	1.22658172	0.00215035	Up
509	ITGB1	1.25405897	0.00127809	Up
510	ITGB8	1.23908417	0.01835046	Up
511	ITPR2	1.40571394	0.01302456	Up
512	JADE1	1.25569944	0.03610671	Up
513	JAG1	1.35466015	0.00126059	Up
514	JAK1	1.35336382	0.00069408	Up
515	JAM3	1.6327523	0.00013647	Up
516	JUN	1.36089705	0.00160003	Up
517	KAZN	0.73815474	0.00767704	Down
518	KCNJ2	0.77138215	0.00936379	Down
519	KCNK12	1.38802658	0.00568643	Up

520	KCNK3	1.21230367	0.00122541	Up
521	KCNMA1	1.40434863	0.00160826	Up
522	KCTD12	1.37016	0.00018836	Up
523	KCTD13	0.82513351	0.0022124	Down
524	KDELR3	1.38895581	0.00119075	Up
525	KIAA0232	0.79265591	0.01113225	Down
526	KIF26B	1.3582067	0.02057253	Up
527	KLF11	1.20522496	0.02845644	Up
528	KLF6	1.47842667	0.00021069	Up
529	KLF7	1.2307221	0.00521509	Up
530	KLF9	1.4496299	0.00021876	Up
531	KPNA2	1.24488757	0.0009522	Up
532	KRAS	1.31942715	0.00744513	Up
533	KSR1	1.21776436	0.02185531	Up
534	LAD1	0.79415353	0.01210832	Down
535	LAIR2	1.72256852	0.00027128	Up
536	LAMA2	1.21244659	0.01297905	Up
537	LAMB1	1.49223859	1.9278E-05	Up
538	LAMP1	1.2721701	0.00083961	Up
539	LAPTM4B	1.41682531	0.00049313	Up
540	LAPTM5	1.21805427	0.0004284	Up
541	LARP4	1.28723811	0.01351066	Up
542	LASP1	1.38042058	0.00277472	Up
543	LDHA	1.35736166	0.0194561	Up
544	LDHB	1.24684862	0.01414104	Up
545	LDLR	1.57085561	2.5242E-05	Up
546	LEP	1.36471361	2.579E-06	Up
547	LEPROT	1.26821237	0.02259359	Up
548	LGALS1	1.43755859	0.00357945	Up
549	LGALS13	0.76365888	0.01820505	Down
550	LGALS3	1.41749664	0.01699396	Up
551	LGALS8	0.78943144	0.01240338	Down
552	LGR5	1.209345	3.558E-05	Up
553	LIFR	1.37303355	0.0027871	Up
554	LIMA1	1.26122596	5.4643E-05	Up
555	LIMK2	0.76376672	0.00562045	Down
556	LMO7	1.26270374	0.00491918	Up
557	LMOD1	1.39891595	0.00890249	Up
558	LNPEP	1.23395457	0.00085789	Up
559	LOX	1.20732336	0.00055325	Up
560	LPAR1	1.57297541	9.9896E-06	Up
561	LPGAT1	1.22443148	0.04948914	Up
562	LPIN1	1.35565933	0.04352812	Up
563	LPP	2.19179023	6.418E-05	Up
564	LRPPRC	1.29740512	0.00381395	Up
565	LRRFIP1	1.38725666	9.2236E-05	Up
566	LRRN3	1.37604926	0.03591251	Up
567	LTF	0.71650438	3.6044E-05	Down
568	LUC7L3	1.20485962	0.00691087	Up
569	LUM	1.38783584	0.00037323	Up
570	LY6G6C	0.7836332	0.00934894	Down
571	LY96	1.26665336	0.02830399	Up

572	LYVE1	1.36473868	2.9299E-06	Up
573	MACF1	1.42746319	0.00420868	Up
574	MAF	1.31983992	7.5006E-05	Up
575	MAFB	1.34604776	0.00013762	Up
576	MAFK	0.7234729	0.00028061	Down
577	MAGEA10	0.8327686	0.00469647	Down
578	MAGEA4	0.78629472	0.00347621	Down
579	MAN1A1	1.60897347	1.1974E-05	Up
580	MAN1C1	0.7253763	0.00881164	Down
581	MAOB	1.3482148	0.00134881	Up
582	MAP1B	1.68621176	4.2567E-06	Up
583	MAP3K5	1.3617123	0.01350008	Up
584	MAPRE2	1.20654602	0.01689797	Up
585	MARCO	1.30027287	0.0228946	Up
586	MAT2A	1.34401603	0.00306332	Up
587	MBNL1	1.33668455	0.00011562	Up
588	MBNL2	1.25039174	0.00509462	Up
589	MBP	1.24018876	0.01947089	Up
590	MC1R	0.82773305	0.00072047	Down
591	MCAM	1.42506118	0.00337916	Up
592	MCC	1.29305389	0.0150787	Up
593	MCL1	1.24794492	0.00174832	Up
594	MCM6	1.29364087	0.01761484	Up
595	MDFI	1.20061358	0.00165989	Up
596	ME2	1.20322977	0.00532372	Up
597	MED13L	1.342848	0.00420722	Up
598	MED6	1.29459778	0.00119838	Up
599	MEF2A	1.20025196	0.04656876	Up
600	MEF2C	1.24809558	0.00184926	Up
601	MEG3	1.6936098	5.5417E-05	Up
602	MEIS1	1.54301226	0.00398959	Up
603	MEIS2	1.33750053	0.00854992	Up
604	METTL7A	1.2468521	0.00062304	Up
605	MFAP4	1.38188008	0.00060427	Up
606	MFGE8	1.32122847	0.00017253	Up
607	MGA	1.32956552	0.00193599	Up
608	MGAT2	1.3155609	0.01120377	Up
609	MGP	1.29654275	0.03109421	Up
610	MICAL2	1.39354706	0.00064014	Up
611	MINPP1	0.69543611	1.7766E-05	Down
612	MITF	1.28688179	0.00014694	Up
613	MKLN1	1.29171356	0.00180098	Up
614	MKNK1	1.38668693	0.00128666	Up
615	MLXIP	1.32313027	0.00171479	Up
616	MME	1.41929291	0.00723083	Up
617	MMP14	1.22461956	0.00243805	Up
618	MMP16	1.2343663	0.00696093	Up
619	MMP19	1.40485783	0.00168309	Up
620	MMP2	1.64271891	0.00033049	Up
621	MORF4L2	1.34516827	0.00195635	Up
622	MPDZ	1.22903113	0.02690056	Up
623	MPHOSPH6	1.21348256	0.02524459	Up

624	MPHOSPH8	1.20526215	0.03592	Up
625	MPZL1	1.35394554	0.000409	Up
626	MR1	1.23609576	0.00322335	Up
627	MRC1	1.36320716	0.00321034	Up
628	MS4A6A	1.24914904	1.5556E-05	Up
629	MSMO1	1.37660854	0.00063201	Up
630	MT1E	1.22025498	0.0008032	Up
631	MT1X	1.24474573	0.00046075	Up
632	MTDH	1.48359106	0.00050402	Up
633	MTHFD2	1.47897754	0.00047656	Up
634	MTMR1	0.74502259	0.00220276	Down
635	MTMR4	0.828012	0.01596227	Down
636	MVB12B	0.75859726	0.00022299	Down
637	MXRA5	0.80848152	0.00255449	Down
638	MXRA7	1.47670077	7.7824E-06	Up
639	MYH10	1.40945039	0.00128441	Up
640	MYH11	1.34710786	1.5457E-06	Up
641	MYL9	1.52927393	0.00449193	Up
642	MYO10	1.36701533	0.00248485	Up
643	NAMPT	1.26704107	0.01819304	Up
644	NAP1L1	1.31844727	0.00052518	Up
645	NAV2	1.42960426	0.00125019	Up
646	NAV3	1.30747792	0.00982682	Up
647	NBN	1.26519172	0.01597042	Up
648	NCKAP1L	1.32556428	5.6626E-06	Up
649	NCOA1	1.4007674	0.01152195	Up
650	NDRG4	0.7888186	0.02289054	Down
651	NEB	0.70978666	1.2688E-05	Down
652	NEDD4L	1.49378411	4.2122E-05	Up
653	NEDD9	1.3700256	0.00212971	Up
654	NEK7	0.80026816	0.02065964	Down
655	NFASC	1.88341193	1.5213E-06	Up
656	NFAT5	2.2340986	6.3963E-06	Up
657	NFE2	0.82539343	0.02575145	Down
658	NFIB	1.28553663	0.0020877	Up
659	NFYB	1.23540615	0.03619542	Up
660	NID1	2.04230628	5.323E-07	Up
661	NID2	1.51872642	0.00099128	Up
662	NKTR	1.64426967	0.00864892	Up
663	NME7	1.32651655	0.01528855	Up
664	NMNAT2	0.82655628	0.00587229	Down
665	NNT	1.21895953	0.00832307	Up
666	NPC2	1.27888788	0.00094044	Up
667	NPL	1.34634261	0.0035575	Up
668	NR2F2	1.22542167	0.02189656	Up
669	NRF1	0.78966576	0.00055634	Down
670	NRIP1	1.40080039	0.00522817	Up
671	NRP2	1.44573777	0.00043816	Up
672	NT5DC2	1.24160168	0.04179785	Up
673	NUAK1	1.54352272	0.00022742	Up
674	NUCKS1	1.30000729	0.00428815	Up
675	NUPR1	1.37654176	0.00098965	Up

676	NUSAP1	1.2612054	0.00152684	Up
677	NXN	1.21719463	0.04514292	Up
678	NXPE3	1.20186962	0.00600467	Up
679	NXPH4	1.219025	0.02900974	Up
680	OAS1	1.21723838	0.00221732	Up
681	OAT	1.37128672	0.02419507	Up
682	ODC1	1.33364751	0.00330912	Up
683	OGN	1.95644517	0.00015945	Up
684	OLFML2B	1.26846004	0.00909902	Up
685	OLFML3	1.54525354	5.8615E-05	Up
686	OLR1	1.2066093	0.00543109	Up
687	OMD	1.41415675	6.9463E-05	Up
688	OPTN	1.23693789	0.00103178	Up
689	OSBPL1A	1.25100672	0.00465275	Up
690	OSBPL3	0.8324888	0.04284257	Down
691	OSBPL8	1.27259362	0.02567489	Up
692	OSER1	0.81364523	0.00771635	Down
693	OSMR	1.24625054	0.03501817	Up
694	OTUB2	1.32961078	0.00029314	Up
695	P2RY1	1.27431272	0.03951324	Up
696	P2RY14	1.39307527	0.00064658	Up
697	P2RY2	0.80757006	0.02729985	Down
698	P4HA1	1.28040049	0.03683261	Up
699	PACSIN3	0.8052425	0.03774698	Down
700	PADI1	1.42409748	0.00070044	Up
701	PAGE4	1.26047691	0.01086978	Up
702	PALLD	1.41008798	0.00334258	Up
703	PANX1	1.25809745	0.01067332	Up
704	PAPPA	0.64822716	0.00227069	Down
705	PAPPA2	1.46979252	2.6564E-06	Up
706	PAPSS1	1.23745575	0.04929356	Up
707	PAPSS2	1.42104085	0.0009858	Up
708	PAQR3	1.2059822	0.04295388	Up
709	PAQR6	0.80285084	0.01307318	Down
710	PARM1	1.33929969	0.01605381	Up
711	PARVA	1.38057109	0.0003957	Up
712	PCBP2	1.25913797	0.0043289	Up
713	PCDH1	0.7375503	0.00172279	Down
714	PCDH17	1.38098028	0.00475329	Up
715	PCOLCE	1.34591256	0.00691087	Up
716	PCSK6	1.40589217	0.00028005	Up
717	PCYOX1	1.23074742	0.00932173	Up
718	PDCD1LG2	1.26074085	0.00182108	Up
719	PDE3A	1.23406388	9.362E-05	Up
720	PDE4D	1.25308565	0.02443003	Up
721	PDGFA	1.2820789	0.03101929	Up
722	PDGFC	1.34187435	5.6018E-05	Up
723	PDGFRA	1.6335307	0.00043329	Up
724	PDIA4	1.21012931	0.01372115	Up
725	PDK4	1.33451297	2.4737E-05	Up
726	PDLIM1	1.25513222	0.010057	Up
727	PDLIM3	1.43853216	0.00016734	Up

728	PDPN	1.4131603	1.9857E-05	Up
729	PDZRN3	1.39912305	0.00012912	Up
730	PEG10	2.78474296	2.4992E-06	Up
731	PELO	1.25023884	0.02537052	Up
732	PF4	1.55000184	0.04188047	Up
733	PFKFB3	1.20348741	0.00214068	Up
734	PFKP	1.30992291	0.00402142	Up
735	PGAP3	0.77449031	0.00988177	Down
736	PGD	1.38438001	0.0063356	Up
737	PGM3	1.29731837	0.00444312	Up
738	PGRMC1	1.26815231	0.00154482	Up
739	PHACTR2	2.18931529	7.8215E-05	Up
740	PHACTR4	1.28113751	0.00377566	Up
741	PHKB	1.23569663	0.04471276	Up
742	PHLDA1	1.31721352	0.00121664	Up
743	PHLDA2	1.33565642	0.00655047	Up
744	PHYH	1.44857325	0.00040777	Up
745	PIGO	0.82145243	0.03785834	Down
746	PIK3CB	1.52946781	0.00180474	Up
747	PIK3R1	1.84016425	0.00200595	Up
748	PIP5K1B	0.78499351	0.00501868	Down
749	PIPOX	1.81537398	2.291E-05	Up
750	PITX2	1.74973419	0.00012469	Up
751	PKN2	1.62072275	0.00017577	Up
752	PLA2G2A	1.86256123	4.1851E-06	Up
753	PLA2G4A	2.24769954	1.4892E-05	Up
754	PLA2G7	1.95727232	2.3481E-06	Up
755	PLAC8	2.19852509	1.2891E-08	Up
756	PLAGL1	1.3748806	0.00025002	Up
757	PLAU	0.82355919	0.02498184	Down
758	PLCB1	0.73196173	0.00773313	Down
759	PLCL1	1.30088651	0.03229389	Up
760	PLCL2	0.79969271	0.00077521	Down
761	PLEK	1.31452268	1.1385E-06	Up
762	PLEKHA6	0.73169687	0.00370034	Down
763	PLEKHF1	0.80272211	0.00371813	Down
764	PLOD2	1.98008672	6.5298E-06	Up
765	PLP2	1.29512906	0.01361995	Up
766	PLTP	1.35976199	0.00263121	Up
767	PLXDC2	1.28898916	0.00420926	Up
768	PLXNC1	1.2638092	0.03240204	Up
769	PMAIP1	1.28626385	6.1865E-05	Up
770	PMP22	1.56881964	3.3123E-05	Up
771	PODXL	1.2174364	0.0361615	Up
772	POF1B	0.81857502	0.00338256	Down
773	POMC	1.216319	0.00080365	Up
774	POMGNT1	0.80353512	0.00389613	Down
775	PON2	1.34815868	0.00054585	Up
776	POSTN	0.77880257	0.0017148	Down
777	PPBP	1.58279943	0.04426627	Up
778	PPFIBP1	1.40907543	0.02458785	Up
779	PPIC	1.25068189	0.00159417	Up

780	PPIG	1.29974596	0.0101383	Up
781	PPP3CA	1.26213702	0.03700027	Up
782	PPP4R4	1.23821015	0.00989228	Up
783	PRDX2	1.29568769	0.0348764	Up
784	PRDX4	1.23270303	0.01011267	Up
785	PRG2	2.33533974	0.00027962	Up
786	PRKAR2A	1.21632404	0.00559039	Up
787	PRKCA	1.35166452	0.00458223	Up
788	PRKCB	1.23424615	0.01381273	Up
789	PRKCI	1.52016373	0.00074837	Up
790	PRKD2	0.77158152	0.00619262	Down
791	PRKD3	1.3204564	0.0264027	Up
792	PRNP	1.22323954	0.00920673	Up
793	PROS1	1.23673423	0.00049105	Up
794	PROSER1	0.83023394	0.02589232	Down
795	PRPS2	1.27672832	5.3466E-05	Up
796	PRR11	1.21362722	0.03450124	Up
797	PRRC2C	2.65215456	3.5986E-06	Up
798	PRRX1	1.22184813	0.00105564	Up
799	PRUNE2	1.62514382	2.2512E-05	Up
800	PSD4	0.79019516	0.01242215	Down
801	PSG1	0.70652733	0.00680856	Down
802	PSG11	0.74971619	0.00394867	Down
803	PSG4	0.78157874	0.00777072	Down
804	PSG9	0.76929262	0.00022495	Down
805	PSMB8	1.22560562	0.00351897	Up
806	PTGER3	1.48624773	0.00464801	Up
807	PTGES	1.45049454	7.9548E-05	Up
808	PTGS2	1.41177148	2.2816E-05	Up
809	PTH1R	1.21193229	0.03391606	Up
810	PTN	1.4459677	0.00019548	Up
811	PTP4A1	1.92932023	2.886E-05	Up
812	PTPN13	1.36700853	0.00046357	Up
813	PTPN3	0.83244462	0.00083423	Down
814	PTPRB	0.83160098	0.00019135	Down
815	PTPRC	1.22797165	0.00047957	Up
816	PTPRE	1.24028191	0.04369407	Up
817	PTX3	1.53372956	0.00012569	Up
818	PVR	1.22342676	0.00059507	Up
819	PXDN	1.6553452	7.9068E-05	Up
820	PYCR1	1.2704388	0.03014135	Up
821	QSOX1	1.20680486	0.00534091	Up
822	RAB20	1.25305788	0.00871954	Up
823	RAB23	1.45147331	0.00064367	Up
824	RAB2A	1.40979531	0.02500676	Up
825	RAB31	1.29983931	0.02802612	Up
826	RAB32	1.20619558	0.0209519	Up
827	RAB36	0.83313985	0.0422085	Down
828	RAB6B	0.80428294	0.00345859	Down
829	RAB8B	1.4723513	0.00173449	Up
830	RABGAP1L	2.1902155	0.00023909	Up
831	RAD23B	1.24169395	0.02419397	Up

832	RALGPS2	1.27293465	0.00192491	Up
833	RAMP1	1.31585478	0.00631344	Up
834	RAP1GAP	0.79788529	0.00667619	Down
835	RAP1GAP2	0.81250294	0.00068325	Down
836	RAP2A	1.33592324	0.00443892	Up
837	RAP2B	1.27311	0.00294244	Up
838	RAPGEF3	0.82425808	0.00234273	Down
839	RASGRP2	1.24919399	0.01818598	Up
840	RB1	1.31366775	0.00240056	Up
841	RBFOX2	1.31708039	0.00302293	Up
842	RBM22	0.76489824	0.00244507	Down
843	RBM39	1.46036077	0.0003233	Up
844	RBP1	1.74702309	6.1865E-05	Up
845	RBPMS	1.21781031	0.00325719	Up
846	RCN3	1.20000727	0.02002321	Up
847	RDX	1.20027669	0.0029586	Up
848	RECK	1.22349878	0.00446417	Up
849	RECQL	1.2099725	0.02445092	Up
850	RECQL5	0.79221528	0.00511426	Down
851	REEP1	1.33418065	0.00170687	Up
852	REL	1.31282248	0.00164533	Up
853	REPS2	1.33578698	0.0003638	Up
854	RGL1	1.21038993	0.00105653	Up
855	RGS10	1.24295362	0.01287275	Up
856	RGS2	1.25447321	0.00314253	Up
857	RGS5	1.20494287	0.00040991	Up
858	RHOD	0.80596003	0.00431585	Down
859	RIN2	1.20965212	0.01844442	Up
860	RNASE4	1.24603002	0.00834303	Up
861	RNASE6	1.37306851	0.00215666	Up
862	RND3	1.29298935	0.01486265	Up
863	RNF114	0.78200621	0.02550459	Down
864	RNF39	0.80567854	0.02463337	Down
865	ROBO1	1.30659026	0.00246994	Up
866	ROCK2	1.27856269	0.02391639	Up
867	ROR1	1.31929996	0.00336846	Up
868	RORA	0.68548874	0.00051722	Down
869	RPL14	1.23734259	0.01150236	Up
870	RPL38	1.24113341	0.01870001	Up
871	RPLP0	1.21262882	0.00098195	Up
872	RPP25	1.24435968	0.00095877	Up
873	RPS21	1.20658367	0.00955421	Up
874	RPS4Y1	1.24844177	5.7485E-05	Up
875	RPS6KA6	0.81249813	0.00854992	Down
876	RPS9	1.20329643	0.0020352	Up
877	RRM2	1.3784081	0.0096492	Up
878	RUFY3	1.47151616	0.00034632	Up
879	RUNX1T1	1.25626471	2.9331E-05	Up
880	S100A10	1.28701756	0.00024801	Up
881	S100A12	1.30709701	0.00539125	Up
882	S100A4	1.33155794	0.0018276	Up
883	S100A8	1.3239449	3.444E-05	Up

884	SAMM50	1.20491091	0.00079884	Up
885	SAP30	1.22115568	0.04560672	Up
886	SASH1	1.29172285	0.00090272	Up
887	SAT1	1.2615047	0.00161962	Up
888	SBSPON	1.32446613	0.0008796	Up
889	SCAF11	1.26533218	0.00640674	Up
890	SCAMP5	1.31814403	0.02057253	Up
891	SCARB2	0.72295089	9.8036E-05	Down
892	SCD	1.60548901	0.00040905	Up
893	SCGN	1.54307266	0.02874437	Up
894	SCN7A	1.33997649	0.00103553	Up
895	SCNN1B	0.73707252	0.00260946	Down
896	SEC14L1	1.2740363	0.00113949	Up
897	SEC23A	1.37323318	0.00758313	Up
898	SECTM1	1.26981888	0.00047543	Up
899	SEH1L	1.32812639	0.00083071	Up
900	SELENBP1	1.27621745	0.00330331	Up
901	SEMA3E	1.26012194	0.02533489	Up
902	SEMA4A	0.8180931	0.02646903	Down
903	SEMA5A	1.6245315	1.5058E-05	Up
904	SEMA6D	1.42614533	8.7769E-05	Up
905	SERPINB6	1.2229981	0.01975229	Up
906	SERPINE1	1.4066594	2.7618E-05	Up
907	SERPINE2	1.46621722	7.2482E-05	Up
908	SERPING1	1.61419309	0.00037186	Up
909	SERPINH1	1.20181577	0.00241064	Up
910	SETBP1	1.23584785	0.01953739	Up
911	SF3B1	1.25381716	0.00743362	Up
912	SFRP1	1.33617317	0.00940906	Up
913	SGPP1	0.75566293	0.00536853	Down
914	SH3BGRL	1.32829653	0.0003683	Up
915	SH3BP2	0.75028462	0.00880811	Down
916	SH3BP5	1.29480778	0.00043474	Up
917	SH3GLB2	0.82853924	0.00988277	Down
918	SHANK2	0.81358704	0.00804294	Down
919	SKAP2	1.22890175	0.00808541	Up
920	SKP2	1.49521094	0.00012087	Up
921	SLA	1.25014024	0.00067942	Up
922	SLC12A8	1.4671863	6.0988E-05	Up
923	SLC16A1	1.23288218	0.00628991	Up
924	SLC16A10	1.40957972	0.00030227	Up
925	SLC16A7	1.4508712	0.00086273	Up
926	SLC1A2	1.24182892	0.00302727	Up
927	SLC1A3	1.26930444	0.0149059	Up
928	SLC22A11	1.24640091	0.01772191	Up
929	SLC22A18	1.31633193	0.03981831	Up
930	SLC22A3	1.3742831	5.8514E-06	Up
931	SLC22A5	0.83018698	0.03481365	Down
932	SLC23A2	0.77840523	0.00066923	Down
933	SLC25A15	1.2567825	0.00983725	Up
934	SLC25A37	1.41241073	0.00471419	Up
935	SLC26A2	1.52821701	4.2625E-06	Up

936	SLC28A2	1.57654574	0.00122016	Up
937	SLC2A10	1.27905101	0.03380111	Up
938	SLC2A3	1.67562495	1.5662E-05	Up
939	SLC34A2	1.28859017	0.02212677	Up
940	SLC35A3	1.26595208	0.00140137	Up
941	SLC35D1	1.48785384	0.00591949	Up
942	SLC35E1	1.20780272	0.00621031	Up
943	SLC39A14	1.62493525	2.6239E-05	Up
944	SLC43A3	1.20409502	0.01862185	Up
945	SLC46A3	1.2497056	0.00268805	Up
946	SLC4A4	0.82294458	0.0375915	Down
947	SLC4A7	1.36949566	0.00028666	Up
948	SLC5A3	1.40099203	0.00762194	Up
949	SLC7A2	0.6037398	2.7631E-06	Down
950	SLC7A5	0.82142407	0.02483776	Down
951	SLC7A7	1.29599596	0.01174531	Up
952	SLC7A8	1.20341278	0.00133196	Up
953	SLC8A1	1.23834004	0.00063079	Up
954	SLIT2	1.55997203	0.00020897	Up
955	SLPI	1.71610547	0.00012996	Up
956	SMAD3	1.35304653	0.00081087	Up
957	SMAD5	1.32177088	0.0140347	Up
958	SMAD6	0.79897377	0.00832307	Down
959	SMC3	1.59245697	0.00011527	Up
960	SMC4	1.23464086	0.00362738	Up
961	SMC5	1.29294665	0.03532201	Up
962	SNAI2	1.70158651	1.2528E-05	Up
963	SNRK	1.29808614	0.00424759	Up
964	SNRNP40	1.20294157	0.02630476	Up
965	SNRNP70	0.80769243	0.03240204	Down
966	SNRPE	1.20035408	0.03835107	Up
967	SOAT1	1.41720299	0.00139873	Up
968	SORBS1	0.77966488	0.02812555	Down
969	SORBS2	1.69822919	0.00115687	Up
970	SOX4	1.34171383	0.04188047	Up
971	SOX5	1.35357689	0.00051288	Up
972	SPAG9	1.22806522	0.03706245	Up
973	SPOCK1	1.29714283	0.00024272	Up
974	SPON1	1.30719207	0.00071917	Up
975	SPP1	1.58262564	0.00012938	Up
976	SPTBN1	1.28008991	0.00478958	Up
977	SPTSSA	1.25361397	0.00770341	Up
978	SPX	0.67634503	6.1813E-05	Down
979	SQLE	1.36601797	0.00158289	Up
980	SRGN	1.2759253	0.0009743	Up
981	SRM	1.27424674	0.02772848	Up
982	SRPRB	1.2069681	0.0029202	Up
983	SRPX	1.52411658	0.00066836	Up
984	SSB	1.24674587	0.00796867	Up
985	SSR1	1.21010109	0.04637874	Up
986	ST18	0.65004677	0.00012479	Down
987	ST3GAL1	0.76537507	0.01819686	Down

988	ST3GAL5	1.32210334	0.0001004	Up
989	STAB2	1.45441243	0.00672702	Up
990	STAT1	1.26717547	0.00273161	Up
991	STAT3	1.24535687	0.00211441	Up
992	STAT4	1.35382833	0.000459	Up
993	STC1	1.91663626	8.3647E-08	Up
994	STEAP4	0.83186265	5.2559E-05	Down
995	STK3	0.81603055	0.01026301	Down
996	STK4	1.20100454	0.04433314	Up
997	STRA6	0.80438516	0.03812664	Down
998	STX2	1.22515479	0.00733544	Up
999	STX6	1.20468201	0.00710192	Up
1000	SULT2B1	0.80622347	0.04131419	Down
1001	SUN1	1.22787435	0.00288032	Up
1002	SUPT16H	1.28120913	0.00233963	Up
1003	SV2B	1.82534387	0.0017834	Up
1004	SVIL	1.36490822	0.00040803	Up
1005	SYNCRIP	1.20165137	0.00518346	Up
1006	TAC3	2.40533235	1.3157E-10	Up
1007	TACC2	1.38809868	6.0203E-05	Up
1008	TAGLN	1.58172687	0.00764792	Up
1009	TAOK1	1.45481327	0.0006676	Up
1010	TAOK3	1.25900573	0.00268135	Up
1011	TAP1	1.33921291	0.00488362	Up
1012	TBC1D1	0.82575178	0.00899636	Down
1013	TBL1X	1.29074036	0.01130741	Up
1014	TCEAL4	1.31735178	0.00137542	Up
1015	TCF7L2	1.60754838	8.7913E-06	Up
1016	TEAD1	1.24612886	0.00153957	Up
1017	TENM3	1.31387232	0.00552608	Up
1018	TFAM	0.80526091	0.02430445	Down
1019	TGFB2	1.45816865	0.00128969	Up
1020	TGFB3	1.53493373	0.00165023	Up
1021	TGFBI	1.31527151	0.00108997	Up
1022	TGFBR1	1.20978024	0.00291385	Up
1023	TGFBR3	1.30989857	0.03641657	Up
1024	TGIF1	1.29222429	0.00258718	Up
1025	THBS1	1.91404187	0.00185441	Up
1026	THSD4	0.81719722	0.00044507	Down
1027	THSD7A	0.76737408	1.1641E-07	Down
1028	THY1	1.52011163	0.00041603	Up
1029	TIMP1	1.40294477	0.00038799	Up
1030	TIMP3	1.69782958	5.5675E-07	Up
1031	TIMP4	1.26535498	0.03757425	Up
1032	TIPARP	1.29660105	0.00238992	Up
1033	TLE4	1.20373494	0.00091354	Up
1034	TMED2	1.48325919	0.00063555	Up
1035	TMEM132A	0.79678431	0.00522756	Down
1036	TMEM168	0.76314513	0.0125258	Down
1037	TMEM45A	1.93778916	2.3034E-06	Up
1038	TMEM50B	1.26675203	0.00287395	Up
1039	TMEM74B	0.77959656	0.00582642	Down

1040	TMEM97	1.26659732	0.0206807	Up
1041	TMOD3	1.21145872	0.02362852	Up
1042	TMX1	1.46702129	0.00030138	Up
1043	TNFRSF1B	1.24513937	0.01655023	Up
1044	TNFSF10	1.32591866	0.00147719	Up
1045	TNKS2	1.28820199	0.0247708	Up
1046	TNNT1	1.31203828	0.01355987	Up
1047	TNNT3	0.77471194	0.00079696	Down
1048	TNRC6B	1.29310194	0.0021331	Up
1049	TOB1	1.50889942	0.00050782	Up
1050	TOP1	1.24299379	0.00201931	Up
1051	TOP2A	1.35572685	0.00107617	Up
1052	TPD52	0.75063364	0.01071702	Down
1053	TPM1	1.72925189	4.1145E-06	Up
1054	TPM2	1.63368875	0.002636	Up
1055	TPPP3	0.71620254	0.00031208	Down
1056	TPR	1.3353342	0.00457735	Up
1057	TRAF5	1.30348233	0.00866136	Up
1058	TRAK2	1.2923406	0.02031575	Up
1059	TREM1	1.52716137	0.00917186	Up
1060	TRIM14	1.34126297	0.00876384	Up
1061	TRIM45	0.73258545	0.00029572	Down
1062	TRIM62	0.81441908	0.04038905	Down
1063	TRMT13	1.30418414	0.02973151	Up
1064	TRPC4	1.274252	0.00273307	Up
1065	TRPV6	0.79085426	0.02298628	Down
1066	TSPAN13	1.29843825	0.00457486	Up
1067	TSPAN5	1.21448121	0.00145841	Up
1068	TSPAN6	0.79283311	0.02420584	Down
1069	TSPYL5	1.21982579	0.00083423	Up
1070	TUBAL3	0.82573975	0.04627966	Down
1071	TUBB1	1.44576998	0.04545886	Up
1072	TUBB2A	1.57415164	0.00015005	Up
1073	TUBB6	1.38348708	0.00013935	Up
1074	TUSC3	0.78890007	0.03619277	Down
1075	TWF1	1.24102829	0.0018276	Up
1076	TWIST1	0.72065533	0.0020466	Down
1077	TWSG1	1.24008422	0.00197131	Up
1078	TXK	0.69561344	0.00127709	Down
1079	TXN	1.20693825	0.02835437	Up
1080	TXNIP	1.50233166	0.00092219	Up
1081	UBE2B	1.27259657	0.00724956	Up
1082	UBE2N	1.2379818	0.00525538	Up
1083	UGCG	1.29212095	0.01300073	Up
1084	UGDH	1.41928774	0.00025177	Up
1085	UGP2	1.40756943	0.03032461	Up
1086	USO1	1.40038216	0.00052952	Up
1087	USP27X	0.76166424	0.00570769	Down
1088	UTRN	1.21259102	0.00989228	Up
1089	VAMP2	0.77899551	0.00549527	Down
1090	VCAM1	1.3027849	0.00016002	Up
1091	VCAN	1.69334639	2.0589E-06	Up

1092	VCL	1.30707089	0.00673637	Up
1093	VEGFA	2.44009462	5.7717E-07	Up
1094	VEGFC	1.21864307	0.02069303	Up
1095	VKORC1	1.22376632	0.01934161	Up
1096	VNN1	1.36386064	0.03452196	Up
1097	VPS13B	1.22167218	0.03272435	Up
1098	VSIG4	1.41797082	0.00014367	Up
1099	VWA5A	0.70082979	0.00334169	Down
1100	WDR1	1.21928663	0.00411374	Up
1101	WDR45	0.81739308	0.02055345	Down
1102	WIPF1	1.28176176	0.01404893	Up
1103	WLS	1.30495048	0.0006756	Up
1104	WNT2	1.6551637	0.0014249	Up
1105	WNT4	1.22466068	0.00473769	Up
1106	WNT5A	1.49950173	0.00025596	Up
1107	WNT7A	1.26126231	0.00286574	Up
1108	WWC1	0.73411374	0.00081729	Down
1109	YAP1	1.2081721	0.00242182	Up
1110	YME1L1	1.33107131	0.0039543	Up
1111	YWHAZ	1.27743995	0.00177455	Up
1112	YY1	1.20405756	0.01739321	Up
1113	ZBTB16	1.29601294	0.00031015	Up
1114	ZBTB18	0.75335982	0.00492311	Down
1115	ZBTB38	1.31507475	0.00147253	Up
1116	ZBTB43	1.28945198	0.04363455	Up
1117	ZEB2	1.51645579	2.3122E-06	Up
1118	ZFHX3	1.26470113	0.01121173	Up
1119	ZFY	0.81817749	0.02745922	Down
1120	ZFYVE16	1.29782061	0.02719245	Up
1121	ZNF330	1.26314321	0.01480872	Up
1122	ZNF362	0.8231276	0.0094873	Down
1123	ZNF451	1.24409109	0.00389109	Up
1124	ZNF507	0.78520792	0.01660365	Down
1125	ZNF516	1.23720409	0.03936692	Up
1126	ZNF652	1.58256553	7.2735E-05	Up
