# Supplementary material for: Early gestational prediction of spontaneous preterm birth using a validated three-protein serum biomarker panel
Source: BMC Med. 2026 Feb 2;24:138. doi: 10.1186/s12916-026-04639-9 (PMC12955166; doi:10.1186/s12916-026-04639-9)
Supplement: Supplementary file 2 — Supplementary Material 2. [file 12916_2026_4639_MOESM2_ESM.pdf]

	Gene
1	PDS5B
2	DDX60
3	KRT2;KRT4;KRT5;KRT6A;KRT6B;KRT72;KRT75;KRT76;KRT77
4	HTRA4
5	LPA;PLG
6	MAN2A1
7	KRT2
8	A1BG
9	SERPIND1
10	KRT5
11	KRT1;KRT2
12	OBSCN
13	KRT10
14	APOC3
15	SERPINB2
16	SPARC
17	C1S
18	LAMA2
19	ETF1
20	IGLC7
21	APOB
22	ADGRG6
23	PF4
24	ALDOB
25	APOC1
26	KRT9
27	PSG1;PSG3;PSG5;PSG6;PSG7;PSG9
28	ACTA2;ACTB;ACTG1;POTEE
29	SH3BGRL3
30	APOC4
31	SVEP1
32	APOE
33	KRT1
34	LAMC1
35	PFN1
36	P0DOX7__NA
37	TIMP2
38	KRT1;KRT2;KRT77
39	IGHA1;IGHA2
40	CDC5L
41	CPN1
42	FAM20C
43	TREML1
44	LAMP2
45	FLT4
46	DPEP2
47	COL18A1
48	LNPEP
49	CDK5RAP2;PDE4DIP
50	LYZ
51	KRT14;KRT17

52	SPINT1
53	VIL1
54	B2M
55	KRT4;KRT80
56	GPNMB
57	PLA2G7
58	GP5
59	CFP
60	FBLN1
61	KRT10;KRT13;KRT14;KRT16;KRT17;KRT19;KRT24;KRT31;KRT
62	MEGF9
63	PEPD
64	FAM161B
65	CD59
66	KRT2;KRT76
67	FLNA
68	NRCAM
69	TRIM36
70	PSG3;PSG5;PSG6;PSG9
71	PSG1;PSG11;PSG2;PSG5
72	NID1;NID2
73	AHSG
74	SELL
75	EFEMP1
76	DCC
77	IGLL5
78	ANPEP
79	ENPP2
80	PLXNB2
81	MRC1
82	ACTB;ACTG1
83	ADAM12
84	PPBP
85	C2
86	SPEGNB
87	THBS1
88	LAMP1
89	FGB
90	ACTA2;ACTB;ACTG1
91	PCYOX1
92	IGKV3D-20
93	PSG11
94	KRT2;KRT5;KRT6A;KRT6B;KRT75;KRT76
95	GRID1
96	CNDP1
97	ARID5B
98	FGG
99	VNN1
100	C8B
101	KRT72
102	CST3
103	LCAT

104 OIT3
105 CENPF
106 HYAL1
107 C7
108 HBB;HBD
109 ENPEP
110 IGLV7-43;IGLV7-46
111 KRT14
112 C1QC
113 ORM2
114 F7
115 SERPINA4
116 C1QA
117 FRMPD1
118 PRDX2
119 GSR
120 APOF
121 THBS4
122 IGHA2
123 PPIA
124 F9
125 KRT10;KRT14;KRT16;KRT17;KRT19
126 SERPINF1
127 ITIH4
128 MMP9
129 TNFRSF19
130 PF4;PF4V1
131 IGKV3-11;IGKV3D-11
132 GATAD2A
133 PTPRG
134 PSG3;PSG5;PSG9
135 RUFY2
136 MAN2A1;MAN2A2
137 SYNPO2
138 LTA4H
139 IGKV3-7
140 ZNF469
141 SDC1
142 ISM2
143 AOX1
144 GLIPR2
145 HP;HPR
146 NIPAL4
147 LYPD3
148 JCHAIN
149 CD44
150 S100A9
151 ICAM1
152 KRT13
153 ACTB;ACTG1;POTEE
154 IL1R2
155 CFI

156 FCGBP
157 SERPINA1
158 CETP
159 PAPPA
160 TPM4
161 IGHA1
162 CPN2
163 MSN
164 F12
165 C4BPB
166 NOTCH2
167 CFHR5
168 TPI1
169 IGHG1;IGHG3
170 VCL
171 WDR87
172 PSG1;PSG6;PSG9
173 IGHG2;IGHG3;IGHG4
174 PSG1
175 PSG4
176 ITIH1
177 CFHR2
178 ERAP1
179 A2M
180 PSG9
181 SPARCL1
182 AGT
183 PGLYRP2
184 ROBO4
185 FCGR3A
186 ERAP2
187 CFAP69
188 IGFBP3
189 KRT6A;KRT6B
190 Biognosys|iRT-Kit_WR_fusion__NA
191 C4B
192 PROZ
193 VASN
194 SEMA4B
195 IGKC
196 SNED1
197 HLA-A;HLA-C;HLA-H
198 NOTUM
199 KRT16
200 ANG
201 KNG1
202 SERPINA6
203 HRNR
204 APOL1
205 SOD3
206 COMP;THBS4
207 GRN

208 IFFO1;IFFO2
209 PODXL
210 SSC5D
211 PSG3
212 C9
213 IGHV3-74
214 IGHG1;IGHG2;IGHG3
215 SHBG
216 KIT
217 SERPINF2
218 PCSK9
219 CTSZ
220 HEXB
221 PLXDC2
222 NCAM2
223 IGKV4-1
224 CFHR1;CFHR2
225 SLPI
226 ALB
227 HBB
228 C17orf75
229 PSG2
230 SCGB3A1
231 APOM
232 CFD
233 IGHV3-15;IGHV3-72;IGHV3-73
234 SERPINA3
235 NID1
236 CD93
237 PTPRF
238 IGLV1-47
239 MPO
240 MASP2
241 HPX
242 RNASE1
243 MCAM
244 LRRC70
245 PROC
246 PSAP
247 ITIH2
248 MINPP1
249 KRT6A;KRT6B;KRT75
250 TFRC
251 BCHE
252 FETUB
253 CCDC85A
254 ICOSLG
255 IGF1
256 COMP
257 S100A8
258 NCAM1
259 NUTM2A

260 GNPTG
261 RBP4
262 CD96
263 HGFAC
264 SLFN5
265 IGHG1;IGHG2;IGHG3;IGHG4
266 C1R;C1RL
267 H4C1
268 GPLD1
269 LRP1
270 ITGA2
271 C4A
272 CSF1R
273 CFHR1;CFHR5
274 IGHV3-30;IGHV3-30-5
275 GGH
276 QSOX1
277 WDR64
278 CTSF
279 PRKCSH
280 HABP2
281 ECM1
282 TENM3
283 CTBS
284 F10
285 DKK3
286 CTSS
287 MMP2
288 CPQ
289 LGALS3
290 XPNPEP2
291 SELP
292 CTSB
293 MASP1
294 LTBP1
295 SERPINC1
296 LDHB
297 CFHR4
298 FN1
299 ANGPTL3
300 APOC2
301 PRSS2
302 CFH;CFHR1
303 OGN
304 FCN3
305 C1R
306 RELN
307 SELENOP
308 ITIH3
309 PKM
310 SLC3A2
311 CLU

312 CC2D2B
313 IQCA1L
314 C4A;C4B
315 SPDYA
316 EGFR
317 CLEC3B
318 AZGP1
319 DSG2
320 KRT14;KRT16
321 IGFBP1
322 LAMB1
323 PTPRJ
324 VTN
325 PLS1
326 SERPING1
327 LCP1
328 IGFBP2
329 KCNH3
330 AGA
331 IGHG1
332 TTR
333 IGHG2;IGHG3
334 LPA
335 APOA1
336 TAX1BP1
337 TIE1
338 FAM83C
339 NRP1
340 ALCAM
341 F13A1
342 DSP
343 VCAM1
344 APCS
345 FAM135B
346 MMRN2
347 P54802__NAGLU
348 MYH9
349 PVR
350 CFB
351 SPP2
352 CDH18
353 PTPRM
354 CD248
355 APOA2
356 ICAM2
357 GPX3
358 FBN1
359 FSTL3
360 CFH;CFHR3
361 PIGR
362 C5
363 PON1

364 DPP4
365 BASP1
366 ACTBL2
367 FAH
368 ABI3BP
369 C1QB
370 IGF2R
371 C1RL
372 OLFM1
373 KLKB1
374 COL6A1
375 IGKV3-20;IGKV3D-20
376 PROS1
377 IGLV1-51
378 AMBP
379 ESAM
380 IGLV1-40
381 CA1
382 IGF2
383 MST1
384 LILRA1;LILRA3
385 B3GNT2
386 TIMP1
387 OAF
388 ZZEF1
389 GC
390 CDH1
391 CD163
392 APOD
393 PRG4
394 TF
395 TLN1
396 RAB39A
397 IGKV1-27;IGKV1-8
398 FGA
399 CRP
400 CFH
401 AFM
402 CNTN1
403 IL1RAP
404 CST6
405 MERTK
406 APOH
407 HRG
408 PLTP
409 ACE
410 VWF
411 IGKV2-30
412 CDH13
413 APOA4
414 FCN2
415 INHBC

416 IGHG2
417 IGKV1-33;IGKV1D-33
418 FSTL1
419 PCOLCE
420 APP
421 INTS8
422 PDIA3
423 IGKV2-24;IGKV2D-24
424 F2
425 F11
426 CD14
427 PROCR
428 P0DOX2__NA
429 IGLC2;IGLC3
430 C3
431 KLRG1
432 GP1BA
433 C8G
434 SAA1;SAA2
435 LGALS1
436 H6PD
437 C4BPA
438 CFHR3;CFHR4
439 ADIPOQ
440 PSMB6
441 CAMP
442 CAT
443 SERPINA5
444 PRG2
445 IGFALS
446 BTD
447 PLG;PLGLB1
448 MTHFD1
449 CERT1
450 CHL1
451 MET
452 C8A
453 C6
454 CRTAC1
455 MENT
456 ADAMTS13
457 TNXB
458 CILP2
459 PTGDS
460 PZP
461 LBP
462 CBLN4
463 SERPINA10
464 IGHM
465 B4GALT1
466 MAN1A1
467 PLG

468 IGFBP4
469 CDH5
470 LUM
471 DBH
472 SERPINA7
473 HSPG2
474 ZFYVE1
475 SELENBP1
476 GSN
477 F5
478 HSPA5
479 ADAMTSL4
480 LILRA3
481 AOC3
482 HSP90B1
483 POSTN
484 LGALS3BP
485 LYVE1
486 AOC1
487 ADAMTSL2
488 CRYBG2
489 IGFBP5
490 TNC
491 CSH1;CSH2
492 F13B
493 IGKV2-28;IGKV2-40;IGKV2D-28;IGKV2D-40
494 ATRN
495 SRGN
496 MMRN1
497 COLEC10
498 LRG1
499 SAA4
500 IL6ST
501 CPB2
502 CP
503 BNC2
504 COL6A3
505 CD5L
506 PON3
507 PF4V1
508 FUCA2
509 IGKV2-30;IGKV2D-30
510 SAA1
511 ALDOA
512 A2M;PZP
513 APMAP
514 IGKV3D-15
515 TGFBI
516 LTF
517 MBL2
518 IGFBP6
519 MMP3

520 MEGF8
521 ITGB1
522 CACNA2D1
523 AFP
524 FUCA1
525 BPNT2
526 NOTCH3
527 SBSN
528 CD109
529 GSTO1
530 IGKV3-20
