# Supplementary material for: Early gestational prediction of spontaneous preterm birth using a validated three-protein serum biomarker panel
Source: BMC Med. 2026 Feb 2;24:138. doi: 10.1186/s12916-026-04639-9 (PMC12955166; doi:10.1186/s12916-026-04639-9)
Supplement: Supplementary file 3 — Supplementary Material 3. [file 12916_2026_4639_MOESM3_ESM.pdf]

	Symbol
1	ABI3BP
2	ACTA2
3	ACTB
4	AGA
5	ALCAM
6	ANPEP
7	AOC1
8	AOC3
9	APOD
10	APOE
11	APP
12	B4GALT1
13	BNC2
14	C1QB
15	C1R
16	C1RL
17	C1S
18	C2
19	C3
20	CAT
21	CD163
22	CD248
23	CD44
24	CETP
25	CFD
26	CFH
27	CLU
28	COL6A1
29	COL6A3
30	CTSB
31	DPP4
32	EFEMP1
33	ENPEP
34	ENPP2
35	ERAP2
36	F5
37	FBLN1
38	FBN1
39	FCGBP
40	FLT4
41	FN1
42	FSTL3
43	GGH
44	GPNMB
45	GPX3
46	HBB
47	HSPG2
48	IGF1
49	IGFBP2
50	IGFBP3
51	IGFBP5

52	IGKC
53	IL1R2
54	IL6ST
55	ITGB1
56	LAMA2
57	LAMB1
58	LAMP1
59	LDHB
60	LGALS1
61	LGALS3
62	LNPEP
63	LTF
64	LUM
65	LYVE1
66	MAN1A1
67	MCAM
68	MINPP1
69	MMP2
70	MRC1
71	NID1
72	OGN
73	PAPPA
74	PCOLCE
75	PCYOX1
76	PF4
77	PLA2G7
78	PLTP
79	PLXDC2
80	PODXL
81	POSTN
82	PPBP
83	PRDX2
84	PRG2
85	PROS1
86	PSG1
87	PSG11
88	PSG4
89	PSG9
90	PVR
91	QSOX1
92	S100A8
93	SELENBP1
94	SERPING1
95	SLPI
96	SRGN
97	TENM3
98	TGFBI
99	THBS1
100	TIMP1
101	VCAM1
102	VCL
103	VNN1

104 CGA
105 EBI3
106 FLT1
107 LEP
108 PAPPA2
109 PTX3
110 VSIG4
