# Supplementary material for: Early gestational prediction of spontaneous preterm birth using a validated three-protein serum biomarker panel
Source: BMC Med. 2026 Feb 2;24:138. doi: 10.1186/s12916-026-04639-9 (PMC12955166; doi:10.1186/s12916-026-04639-9)
Supplement: Supplementary file 4 — Supplementary Material 4. [file 12916_2026_4639_MOESM4_ESM.pdf]

Sample numbers across gestational-age bins and trimesters in four validation cohorts.

Cohort\GA bin	<16 w	16–20 w	>20 w	T1 (≤13 + 6 w)	T2 (14–27 + 6 w)	T3 (≥28 w)	Total samples (n)
UAB (USA)	n = 5	n = 15	n = 30	n = 4	n = 30	n = 16	n = 50
Stanford (USA)	n = 45	n = 20	n = 62	n = 30	n = 61	n = 36	n = 127
Asia 1 (Hangzhou)	n = 54	n = 18	n = 236	n = 21	n = 167	n = 120	n = 308
Asia 2 (Shenzhen)	n = 29	n =0	n = 44	n = 29	n = 34	n = 10	n = 73

Footnotes:

1. Each entry denotes the number of serum samples analyzed by protein analysis within the specified gestational-age window.
2. Trimester boundaries follow standard obstetric convention: T1 ≤ 13^{+6} weeks; T2 14–27^{+6} weeks; T3 ≥ 28 weeks.
3. Participants contributing longitudinal samples are counted once per time window in which a sample was collected.
